# Boosting Electrochemical Capacitive of Ultrathin Shower‐Pouf Birnessite‐Type MnO_2_ for Porous High‐Mass‐Loading Energy Storage Device

**DOI:** 10.1002/advs.202513473

**Published:** 2025-10-05

**Authors:** Shijin Zhu, Chenxin Zhang, Minghao Sun, Fangdie He, Yuxin Liu, Xinyi Guo, Mengwei Yao, Meng Zhao

**Affiliations:** ^1^ School of Materials and Chemical Engineering Chuzhou University Chuzhou 239000 China

**Keywords:** birnessite MnO_2_, crystal regulation, energy density, high‐mass‐loading electrode, supercapacitor

## Abstract

High‐mass‐loading electrodes are critical for the economic viability and practicability of energy storage devices. Here, shower‐pouf‐like birnessite (SPB) with dense‐core‐free nanostructures are synthesized via a cost‐effective strategy, which significantly reduces the content of electrochemical dead mass caused by insufficient ion diffusion. Ascribed to the F^−^ (from NH_4_F) adsorbed on the (001) crystal plane of birnessite, the thickness of ultrathin birnessite films is regulated to ≈3 nm, providing abundant electrochemical active sites. By constructing high‐speed electronic transfer routes with conductive carbon black (CCB) and carbon nanotubes (CNTs), an SPB/CCB/CNTs electrode delivers a high capacitance of 278.6 F g^−1^ with an optimal high mass loading of 15.3 mg cm^−2^. Based on the optimized slurry, the power‐law *b*‐values, Ohmic resistance and cycling stability can be improved dramatically with increasing mass loading. Hence, a high‐mass‐loading asymmetric supercapacitor, assembled by SPB/CCB/CNTs nanostructure and hierarchical porous carbon composites (HPC/CCB/CNTs), delivers a capacitance of 1.75 F cm^−2^ and a record areal energy density of 0.97 mWh cm^−2^ (39.6 Wh kg^−1^). Moreover, A PV‐SC (photovoltaic‐supercapacitor) system driven mechanical vehicle achieves a travel distance of 4.8 m after being charged for 60 s in sunlight, highlighting the practical application prospect of SPB based high‐mass‐loading electrodes.

## Graphical abstract

Ultrathin Shower‐pouf Birnessite‐type MnO_2_ (thickness: ~3 nm) has been fabricated via the specific adsorption of fluoride ions on the (001) crystal plane of birnessite. By constructing an electrically conductive network and optimizing electrode slurry concentration, the electrode mass loading can be substantially improved. These electrodes also exhibit excellent capacitive properties, thereby being suitable for high‐mass‐loading energy storage devices.

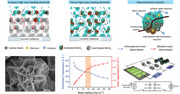

## 1 Introduction

With the rapid development of renewable energy technology, energy storage devices have become increasingly crucial components in smart grids, electric vehicles, and wearable electronics.^[^ ^1^, ^2^, ^3^ ^]^ Supercapacitors, as a potential candidate, have attracted widespread attention owing to their equilibrium between energy density and power density, which is essential property in energy conversion and storage systems.^[^ ^4^, ^5^ ^]^ As a subspecies of MnO_2_, birnessite has been deemed a promising material for supercapacitors owing to its unique layer structure, offering abundant electrochemically active sites and unhindered ionic transfer routes. However, only 20–30% of its theoretical capacitance can be achieved for most birnessite‐based electrodes, which is attributed to the poor electronic conductivity (10^−6^ S m^−1^) that results in a thin active layer (TAL, 1–2 nm) available for energy storage.^[^ ^6^, ^7^ ^]^ In other words, there are a large number of electrochemically inactive regions (dead mass), which occupy a certain mass but contribute less to capacitance.

To eliminate dead mass, birnessite should be designed to be a highly conductive material. Some research has attempted to improve the charge transfer efficiency by coupling with highly conductive matrices, including metal foams, carbonaceous materials and conducting polymers.^[^ ^8^, ^9^, ^10^, ^11^, ^12^, ^13^ ^]^ However, the intrinsic conductivity of birnessite has not been significantly improved, resulting in limited improvements of electrochemical properties. In other words, the thickness of the TAL used for energy storage on the surface of birnessite cannot be extended efficaciously by coupling with highly conductive matrices.^[^ ^14^, ^15^ ^]^ In detail, Mn primarily exists as Mn^4+^ (electronic configuration: [Ar]3d^3^) in an MnO_6_ octahedral coordination environment. After crystal field splitting, the 3d orbitals of MnO_2_ and the 2p orbitals of O form a band gap of 1.64 eV.^[^ ^16^ ^]^ It is difficult for electrons to jump to the conduction band at room temperature, resulting in an extremely low intrinsic carrier concentration. Furthermore, the 3d orbitals of Mn exhibit high spatial locality and weak coupling with the 2p orbitals of O, generating small polarons. The hopping migration of these small polarons requires overcoming energy barriers and is affected by lattice scattering, leading to an extremely low carrier mobility. In addition, the interlayer electron barriers in layered birnessite‐MnO_2_ further hinder carrier transport, which exacerbates the low electrical conductivity of birnessite. Based on this, researchers further attempted to modify the band structure of birnessite by doping method or oxygen vacancy engineering to enhance its intrinsic electrical conductivity and the number of active sites.^[^ ^17^, ^18^ ^]^ However, these approaches do not markedly enhance electrochemical performance, mainly owing to the oversight of the thickness of birnessite films and its internal structures. In most cases, birnessite tends to grow into a rose‐like architecture featuring a dense core, driven by the dissolution‐recrystallization mechanism coupled with disparities in ion diffusion rates. Electrolyte ions exhibit poor diffusivity into the dense inner core and thus fail to electrochemically activate this area.^[^ ^19^, ^20^, ^21^ ^]^ Hence, an indirect strategy to reduce dead mass is to compress the thickness of birnessite films and eliminate the internal dense core. This will dramatically improve the exposure of active units (MnO_6_ octahedra) and thus enhances the mass ratio of TAL in birnessite, thereby generating considerable capacitance.

For practicability, the active material mass loading on electrodes should be high enough (>10 mg cm^−2^), which will significantly reduce the mass ratios of current collector, electrolyte and packaging.^[^ ^22^, ^23^ ^]^ It is widely acknowledged that high mass loading of active materials within the electrode will give rise to a substantial increase in the dead mass ratio. Specifically, the active mass ratio should remain almost unchanged during the energy storage process even with increasing mass loading, due to the adequate total electrical conductivity of the active materials.^[^ ^24^, ^25^ ^]^ However, with increasing active material mass loading, these active materials far away from the current collector tend to disconnect from the main part in terms of electronic transport, ascribed to the inevitable inadequate electronic transmission, resulting in electrochemically inactive regions. Additionally, the electrochemical stress generated from charge accumulation causes irreversible breakage of conducting networks within the active materials, resulting in new dead zones in the electrode.^[^ ^26^, ^27^ ^]^ Thus, both absolute and relative content of dead mass increase in high‐mass‐loading electrodes. Obviously, the charge transfer pathways and ion transport routes become more complex, causing an irreversible degradation of electrochemical properties and resulting in an unbridgeable gap between laboratory research and industrialization.^[^ ^28^, ^29^ ^]^ Furthermore, a large number of closed pores generated by crosslinked birnessite also contribute a nonnegligible amount of dead mass to the electrode because of the existence of binder (PVDF). Thus, both the structure of birnessite and the resulting working electrode should be rationally designed for high‐mass‐loading electrodes to ensure high TAL content, high charge transfer and ion transport efficiency.^[^ ^30^, ^31^ ^]^ However, most current studies are concentrated on the engineering manipulation of birnessite materials, but ignore correlation between the electrochemical performance influenced by microstructures and the structural evolution during electrode fabrication, which is the key breakthrough point for fabricating high‐mass‐loading electrodes.

Herein, SPB nanostructures composed of ultrathin birnessite films with dense‐core‐free structure were fabricated as active materials for high‐mass‐loading electrode. After constructing high‐speed electronic transfer routes by CCB and CNTs, an optimal active material (SPB/CCB/CNTs) slurry concentration of 30 wt% was identified to balance the capacitance and mechanical stability of the electrode. On this basis, a high electrochemical capacitance of 278.6 F g^−1^ with an optimal mass loading of 15.3 mg cm^−2^ was obtained. In addition, the *b*‐values, internal resistance and cycling performance of the electrodes have been significantly improved. Thus, a high‐mass‐loading asymmetric device assembled with SPB/CCB/CNTs and HPC/CCB/CNTs delivers a record areal energy density of 0.97 mWh cm^−2^ (39.6 Wh kg^−1^). A PV‐SC system composed of solar cells, high‐mass‐loading asymmetric supercapacitors, and a mechanical vehicle achieved a travel distance of 4.8 m after being charged for 60 s under sunlight. This remarkable performance highlights the practical application prospects of SPB‐based high‐mass‐loading electrodes.

## 2 Results and Discussion

**Figure 1:**
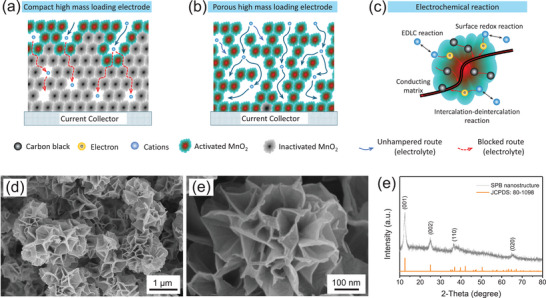
Schematic illustration for a) compact high‐mass‐loading electrode, b) porous high‐mass‐loading electrode and c) electrochemical reaction, d,e) SEM images and f) XRD pattern of SPB.

For high‐mass‐loading MnO_2_ electrodes, the dead mass content (defined in Supporting Information) of energy storage materials in the electrode should be as low as possible, which not only ensures high utilization of active materials but also enhances the total energy storage efficiency of the energy storage device. In general, the loading amount of the active material on the electrode surface should be sufficiently high to generate considerable practical capacitance value. However, dead mass often arises from components that are disconnected from the current collector. Specifically, some MnO_2_ exists in bulk or agglomerated form within the electrode, resulting in inadequate electrochemical reactions with the electrolyte, which is even more serious for compact high‐mass‐loading electrodes. As shown in **Figure** **1**a, the closed pores in the electrode cannot be infiltrated by electrolyte sufficiently, due to the lack of electrolyte infiltration channels. For some semi‐closed holes, the electrolyte cannot be transferred in a timely manner, bring about an accumulation of electrolyte ions in the semi‐closed holes. Thus, in accordance with the Nernst equation,^[^ ^32^ ^]^ the electrode potential tends to approach the boundary of the electrode's working window, thereby inducing inadequate electrochemical reactions.

(1) $$\mathrm{Mn}O_{2}+M^{n+}+ne^{-}=\mathrm{MnOOM}$$

(2) $$\varphi =\varphi^{o}+\frac{RT}{nF}\ln\frac{\gamma_{O}c_{O}}{\gamma_{R}c_{R}}$$

where M is alkali metal ion (Na^+^, K+, Ca^2+^, etc.); *φ* is electrode potential; *φ^o^* is standard electrode potential; *c*_O_ is concentration of oxidized species; *γ*_O_ is activity quotient of oxidized species; *c*_R_ is concentration of reduced species; *γ*_R_ is activity quotient of reduced species. In addition, the poor electrical conductivity of MnO_2_ exacerbates the issue. Charges generated from electrochemical reactions cannot be promptly transferred to the current collector, resulting in charge accumulation that impedes subsequent electrochemical reactions. Thus, compact high‐mass‐loading MnO_2_ electrodes typically suffer from a high dead mass content. As a result, even for MnO_2_ nanostructures with a high specific surface area (SSA), only 20–30% of their theoretical capacitance can be utilized.

The service efficiency of high‐mass‐loading MnO_2_ electrodes can be enhanced by engineering a loose structure during the coating process. Consequently, the content of closed and semi‐closed pores within the electrode can be sufficiently reduced. With improved electrolyte infiltration, electrolyte ions are enabled to migrate into all pores, ensuring that the entire MnO_2_ phase is fully activated by the ions (Figure 1b). Additionally, the absence of excessive electrolyte ion accumulation on the MnO_2_ surface ensures stable fluctuations in the electrode potential, which sufficiently facilitates the electrochemical reaction. Furthermore, an additional electron‐conducting matrix comprising conductive carbon black (CCB) and carbon nanotubes (CNTs) should be constructed (Figure 1c). The charges generated from the electric double layer capacitor (EDLC) reaction, surface redox reaction, and intercalation‐deintercalation reaction can be collected in a timely manner, thereby enabling an uninterrupted and high‐efficiency electrochemical reaction over the entire potential range. In addition, CNTs can act as a bridge for the collected charges, significantly shortening the charge transfer pathway. Thus, a porous high‐mass‐loading MnO_2_ electrode with a sufficient conducting matrix represents a practical and high‐performance electrode.

Based on the discussion above, a shower‐pouf like birnessite (SPB) nanostructure was fabricated via a F^−^ regulatory strategy. As displayed in Figure 1d, the nanostructure was made up of dozens of ultrathin nanosheets which connect with each other forming a porous and loose structure. The nanosheets were sufficiently thin to preclude the maintenance of their flat morphology, resulting in slight crimping at their edges (Figure 1e). The crystal information of SPB nanostructure was confirmed to be birnessite‐type MnO_2_ (JCPDS No. 80‐1098) by XRD pattern, which features a typical layered structure.^[^ ^33^ ^]^ Based on the Bragg's law (2*d*sinθ = *λ*), the interlayer spacing was calculated as *d* = 0.702 nm, which is essentially consistent with the *d*‐value (0.705 nm) of the (001) crystal plane in the standard card. This indicates that the sample possesses a typical layered structure of birnessite. The diffraction peaks at 25.5°, 36.3°, and 65.6° were detected, indexing to the (002), (110), and (020) crystal planes of birnessite, respectively.^[^ ^34^, ^35^ ^]^ No characteristic diffraction peaks of α‐, β‐, γ‐, and λ‐MnO_2_ were observed, indicating the high purity of the sample (Figure 1f). Furthermore, with the increasing F^−^ content, the diffraction peaks corresponding to the (001) and (002) crystal planes show a significant decrease in intensity, which indicates a reduction in the number of birnessite layers (Figure S1, Supporting Information). These results demonstrate that the birnessite‐type MnO_2_ with ultrathin nanosheet could offer a large amount of electrochemically active site, implying an outstanding energy storage property. The ultrathin nature and high crystallinity of birnessite also significantly enhance its intrinsic electrical conductivity, leading to a thicker TAL. Specifically, the intrinsic conductivity of birnessite is fundamentally attributed to the Mn^4+^─O─Mn^3+^ redox‐mediated electron hopping within layers (double‐exchange mechanism).^[^ ^36^ ^]^ However, interlayer interactions act as barriers for electron transport across layers. As the number of layers decreases, the proportion of interlayer interfaces reduces, allowing electrons to traverse these interlayer barriers less frequently and thereby significantly reducing transport resistance. Concurrently, the high crystallinity of birnessite markedly diminishes carrier scattering.^[^ ^37^ ^]^ These combined effects suggest that SPB exhibits promising electrochemical energy storage properties.

**Figure 2:**
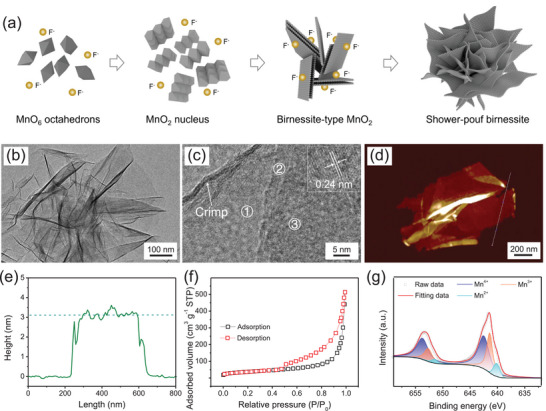
Characterization of SPB: a) schematic representation of crystal growing process, b,c) TEM images, d) AFM image, e) height profiles, f) nitrogen adsorption−desorption isotherms, g) XPS spectra: Mn 2p.

The proposed growth mechanism of as‐prepared SPB nanostructure is illustrated in **Figure** **2**a. Owing to the thermal instability of KMnO_4_, especially in weak acidic solution, a large number of nuclei consisting of several MnO_6_ octahedrons form in the initial stage. These nuclei gradually grow into small birnessite‐type crystals with a typical layered structure. These small birnessite‐type crystals undergo an Ostwald ripening process with small crystals dissolving and recrystallizing onto undissolved crystals. Simultaneously, these undissolved crystals start to connect together by a self‐assembly process to minimize the total surface energy. During the crystal growing process, F^−^ ions are absorbed onto the surface of birnessite slab constituted of MnO_6_ octahedrons based on density functional theory (DFT) calculation results (see Supporting Information). As shown clearly, the adsorption energy of the (001) plane was calculated to be ‐5.26 eV, which is defined as strong adsorption in surface chemistry, indicating the strong interaction between Mn and F atoms. This strong binding enables F^−^ to stably occupy the growth‐active sites of the (001) plane, increasing the vertical growth activation energy and thus suppressing the growth rate. Thus, birnessite preferentially grows along the (100) direction instead of the (001) direction, forming ultrathin nanosheets.^[^ ^38^, ^39^ ^]^ In contrast, the calculated adsorption energies of F^−^ on (100), (010), and (110) planes are >‐0.5 eV (weak adsorption), failing to occupy active sites or alter surface energy, which explains the absence of adsorbed sites on these planes (Figure S2, Supporting Information). Thus, shower‐pouf‐like nanostructures consisting of ultrathin birnessite nanosheets without dense‐core were obtained, ascribing to the competition between the growth of the (100)/(010) planes and the suppressed growth of the (001) plane (Figure 2b). To confirm the ultrathin feature of the birnessite nanosheet, TEM characterization was conducted. Obviously, the dead mass of these porous and dense‐core‐free nanostructures assembled by ultrathin binessite nanosheet is significantly reduced, implying a high exposed rate of MnO_6_ octahedrons. The thickness of these films was calculated to ≈3 nm, indicating approximately 3–4 layers of birnessite slabs (Figures S3 and S4, Supporting Information). In addition, uniform element distribution images of Mn, O, and K was observed for birnessite nanosheets, implying a high structural uniformity of SPB (Figure S5, Supporting Information). Figure 2c displayed the HRTEM images of the ultrathin birnessite films. Clearly, an obvious crimp was observed in HRTEM image, which further confirms the ultrathin feature of the birnessite nanosheets that contain three birnessite slabs. The interplanar crystal spacing was measured to be 0.24 nm, corresponding to the intrinsic spacing of (110) plane. Moreover, three distinct diffraction rings with polycrystal characteristic weredisplayed in SAED pattern (Figure S6, Supporting Information), consisting with (110) and (020) planes, respectively. The ultrathin feature of birnessite has also been detected by atomic force microscope (AFM) (Figure 2d,e and Figure S7, Supporting Information). Obviously, the average thickness of birnessite films was calculated to be 3.2 nm, indicating approximately 3–4 layers of the slabs, which agrees well with the TEM results. These results demonstrate the ultrathin feature of birnessite, resulting in a substantial SSA. The SSA and pore size distribution were measured by Brunauer‐Emmett‐Teller (BET) nitrogen adsorption‐desorption method to further confirm the ultrathin feature of SPB. Obviously, within a relative pressure window of ca. 0.45 to 1.0, a clear hysteresis loop could be observed with the average pore size measured to be 15.2 nm, indicating a fingerprint of mesoporous feature. Combined with the interlayer micropores and macropores generated from the stack of ultrathin birnessite films, the SPB nanostructure provides unhindered ionic transfer routes. In addition, a high SSA of 134.2 m^2^ g^−1^ was obtained for SPB, offering abundant electrochemically active sites. These results indicate the promising electrochemical properties of SPB nanostructures.

The chemical information and components of SPB nanostructure were conducted by XPS (Figure S9, Supporting Information). Clearly, two main Mn 2p peaks were identified, centered at 642.6 eV (Mn 2p_3/2_) and 654.4 eV (Mn 2p_1/2_), attributed to the coexistence of Mn^2+^, Mn^3+^, and Mn^4+^ species.^[^ ^40^, ^41^, ^42^ ^]^ Specifically, the Mn 2p_3/2_ spectrum can be deconvoluted into three peaks at 640.2, 641.47, and 642.56 eV respectively, corresponding to Mn^2+^, Mn^3+^, and Mn^4+^. Meanwhile, the Mn 2p_1/2_ spectrum can also be deconvoluted into three peaks at 651.46 (Mn^2+^), 652.73 (Mn^3+^), and 653.84 eV (Mn^4+^). The calculated spin energy differences were 11.26, 11.26, and 11.28 eV for Mn^2+^, Mn^3+^, and Mn^4+^ respectively (Figure 2g). Based on the contribution of these Mn species, the average valence of Mn was calculated to be 3.59 with the component of birnessite estimated to be K_0.41_Mn^2+^_0.05_Mn^3+^_0.3_Mn^4+^_0.65_O_2_.^[^ ^43^ ^]^ The mixed valence state of Mn offers various energy storage mechanisms, including surface redox process, intercalation–deintercalation process and charge compensation, thereby contributing to a high capacitance of birnessite. The constituent structure of as prepared structure was investigated by Raman spectrometer. As shown clearly in Figure S10 (Supporting Information), only one peak at 635 cm^−1^ was detected in Raman spectrum, corresponding to symmetric stretching vibration of the MnO_6_ group. The absence of deformation modes of Mn─O antisymmetric stretching vibration (575 cm^−1^) and Mn─O bending vibration (498 cm^−1^) can be attributed to the few numbers of birnessite layers. Specifically, the ultrathin birnessite exhibits a lateral dimension of several hundred nanometers with a thickness of merely 3 nm. This structure significantly increases the amount of ordered intra‐layer MnO_6_ octahedra and reduces defects.^[^ ^44^ ^]^ Hence, a significant enhancement of Mn─O symmetric stretching vibration peak was observed. Furthermore, owing to the ultrathin feature (≈3 nm in thickness), the weak asymmetric vibration peaks (500–600 cm^−1^) remain undetected. This can be ascribed to ordered interlayer ions immobilized the orientation of MnO_6_ octahedra and the deformation of ultrathin layers induces inconsistent distortion directions of the octahedra (Jahn‐Teller distortion). These results further demonstrate the ultrathin feature of birnessite nanosheet, generating abundant active sites for electrochemical reactions.

**Figure 3:**
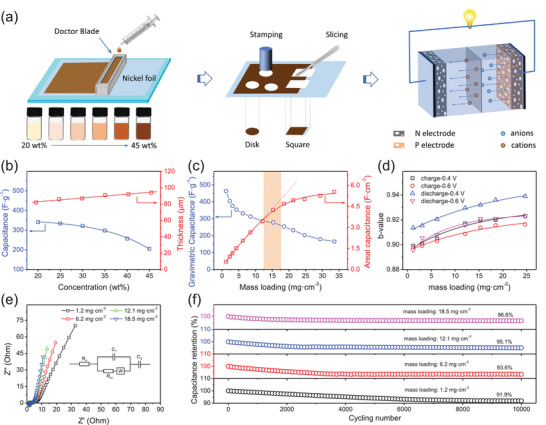
Electrochemical performance of SPB electrode (electrolyte: 1.0 m Na_2_SO_4_): a) electrode fabrication process, b) specific capacitance vibration with slurry concentration, c) capacitance vibration with mass loading, d) vibration of *b*‐values with mass loading at 0.4 and 0.6 V, e) Nyquist plots with an insert of equivalent circuit diagram, f) cycling stability.

To highlight its practical application in high‐mass‐loading electrodes, the electrodes were prepared by a painting method. In detail, SPB nanostructure, CCB and CNTs were mixed at different mass ratios to form a composite slurry. A doctor blade was employed to coat the slurry onto the surface of a nickel foil with a certain thickness (25, 50, 100, 150, 200, 250, 300, 350, 400 µm), which served to control the mass loading of SPB nanostructures. Subsequent to solvent evaporation, the electrode was stamped or cut into various shapes for application in a three‐electrode testing system or energy storage devices (**Figure** **3**a). Obviously, the density of the active material layer is directly proportional to the slurry concentration at a fixed thickness. To determine the optimal mass ratio of conductive additives (CCB and CNTs), a series of electrodes with varying CCB and CNTs contents were fabricated. The optimal mass ratios of CCB and CNTs were identified as 13% and 7.6%, respectively, based on the superior capacitance performance and rate capability (Figure S11, Supporting Information). The method for optimizing the mass ratios of CCB and CNTs was discussed in our previous work.^[^ ^45^ ^]^

The fluctuation of capacitance with the concentration of active materials at a fixed thickness (100 µm) was exhibited in Figure 3b. As shown clearly, a slight increase of thickness was observed with increasing concentration, ascribed to volume shrinkage of the coating. The capacitance of the electrode exhibited an increasingly severe decline, especially after the concentration was increased to 30 wt%. This can be attributed to the increased dead mass, which arises from the reduced pore content caused by solvent evaporation, resulting in insufficient contact between SPB and the electrolyte. Considering the mechanical strength of the coating (with no obvious cracks or delamination) and its 90% mass retention after being pressed under 30 MPa, the optimized concentration was confirmed to be 30 wt% (Figure S12, Supporting Information). Based on the optimal concentration of the slurry, a series of electrodes with varying mass loadings were prepared using a 30 wt% SPB slurry to confirm the optimal mass loading for the electrode (Figure 3c). A high capacitance of 463 F g^−1^ was calculated based on a low mass loading of 1.2 mg cm^−2^, attributed to the ultrathin feature (≈ 3 nm) of SPB, generating abundant electrochemical active sites. A significant damping effect on gravimetric capacitance was observed with the mass loading lower than 5 mg cm^−2^, indicating a decreasing of gravimetric energy density. This can be attributed to the rapid increase in dead mass resulting from blocked electronic/ionic transmission routes and fixed electrochemical reaction depth with increasing mass loading. In general, the dead mass ratio in the electrode remains almost unchanged with the continuous increase in mass loading due to a certain depth of electrochemical reaction. However, the content of unavoidable inactivated mass in the electrode has been considered, which is attributed to unavoidable disconnection with current collectors, although the electronic transfer routes have been improved by CCB and CNTs. Thus, a slight decrease in gravimetric capacitance was observed when the mass loading exceeded 5 mg cm^−2^. This mechanism also results in an almost linear relationship between areal capacitance and mass loading for loadings below 15 mg cm^−2^ followed by a slow increase in areal capacitance with further increases in mass loading. Obviously, the specific/areal energy density changes in accordance with the variation rule of specific/areal capacitance with increasing mass loading. In an ideal scenario, the areal capacitance should increase in direct proportion to mass loading (dotted line in Figure 3c), resulting in an infinite rise in areal energy density and stable gravimetric energy density, regardless of the dead mass in the electrode. Based on the difference between ideal areal capacitance and obtained areal capacitance, the optimized mass loading of 30 wt% SPB/CCB/CNTs slurry in the electrode was confirmed to be 12.5–17.5 mg cm^−2^. Within this range, the actual/ideal ratio on areal capacitance remained above 90%. Hence, with an optimal mass loading of 15.3 mg cm^−2^, an electrode achieves a capacitance of 278.6 F g^−1^, revealing the superiority of high‐mass‐loading SPB electrodes (Table S1, Supporting Information). The enhanced optimal mass loading of SPB/CCB/CNTs electrode can be ascribed to the high conducting network constructed by CCB and CNTs and optimal concentration of slurry, resulting in high electronic/ionic transfer efficiency.

To further investigate the energy storage mechanism of SPB electrodes, the *b*‐values were calculated using the power law equation in CV curves:

(3) $$i=a\cdot v^{b}$$

where *i* (A) and *ν* (V s^−1^) are the measured current and scan rate, respectively. According to the charge storage mechanism of MnO_2_‐based electrode, the current measured from CV curves is contributed by three main contributions: 1) non‐Faradaic contribution from the double‐layer effect; 2) Faradaic contribution involving redox chemistry of surface Mn^3+^/Mn^4+^ ions; and 3) Faradaic reactions referring to Na^+^ intercalation/deintercalation process. The first two contributions (surface capacitive contribution) involve the fast and reversible electrochemical reactions on the surface MnO_2_, which are similar to the EDLC process and cannot be distinguished accurately. Thus, the currents arising from the surface capacitive contribution vary directly with the scan rate, with a corresponding *b*‐value of 1.0. In contrast, the third item is a slow kinetic process with *b* = 0.5, named diffusion‐controlled contribution. Obviously, all the calculated *b*‐values are higher than 0.88, indicating a typically capacitive feature of these SPB electrodes. No obvious redox peaks can be observed in CV curves for all SPB electrodes with all mass loadings. This can be ascribed to the low content of diffusion‐controlled contribution. Specifically, during charge–discharge processes, surface Mn^4+^ ions react with electrolyte ions to achieve rapid charge separation. This charge separation induces surface Mn^3+^/Mn^4+^ redox reactions, generating surface redox capacitance. Meanwhile, owing to the existence of interlayer cations, the associated Mn^3+^ ions (associated with interlayer cations) exhibit diffusion‐controlled capacitance associated with ionic intercalation/deintercalation between interlayers.

With increasing mass loading, the *b*‐values exhibit a slight increase in both the charging and discharging processes. This slight increase indicates a reduced proportion of diffusion‐controlled contribution, which is associated with the low extent of redox reactions of Mn^3+^/Mn^4+^ related to ion intercalation/deintercalation reaction (Figure 3d and Figures S13–S19, Supporting Information). Notable, the increase rate of *b*‐values becomes progressively slower with increasing mass loading, attributed to the reduced proportion of diffusion‐controlled contribution as mass loading increases. Specifically, the surface‐controlled capacitance is more susceptible to inadequate electron transport. Thus, the reduced proportion of diffusion‐controlled contribution in high‐mass‐loading electrode can be ascribed to the efficient electron transport pathways for electrons constructed by CCB and CNTs. Briefly, in high‐mass‐loading electrodes, the capacitance derived from the diffusion‐controlled process is more difficult to manifest compared to that from the surface capacitive process, resulting in high *b*‐values. In detail, for low‐mass‐loading electrodes, SPB particles can be uniformly dispersed within the electrode with large spacing between them, allowing almost the entire surface and shallow interior of all particles to come into contact with the electrolyte. Thus, electrolyte ions can directly diffuse to the active sites on the particle surface, enabling full redox reaction of Mn^4+^/Mn^3+^, including surface capacitive contribution and diffusion‐controlled contribution. Meanwhile, for high‐mass‐loading electrodes, the active sites inside the electrode cannot participate in diffusion‐controlled reactions because ions are “inaccessible,” which corresponds to the linear decay of diffusion‐controlled capacitance with increasing depth. However, the variation of *b*‐values for electrode with mass loadings from 1.2 to 24.7 mg cm^−2^ is inconspicuous, owing to the low content of diffusion‐controlled contribution for all electrodes. The reduced content of diffusion‐controlled contribution can also be verified by electrochemical impedance spectroscopy (EIS), reflected from the linearity region. Clearly, a more verticality can be obtained with increasing mass loading, indicating an ideal capacitive feature (Figure 3e). The values calculated from the equivalent circuit diagram are listed in Table S2 (Supporting Information). Significantly, a slight attenuation of interfacial charge transfer resistance (*R*_ct_) can be found with increasing mass loading, owing to the increased electrochemical active sites within a unit area. However, this value increases to 1.69 Ω with the mass loading of 18.5 mg cm^−2^, implying a high mass transfer resistance. Moreover, high‐mass‐loading electrodes exhibit lower ohmic internal resistance, which is attributed to their interwoven electrical conducting routes (CCB and CNTs). In other words, a high mass loading of active materials enables more effective connections between charge transfer pathways, resulting in reduced ohmic resistance. However, due to the increased number of electrochemically inactive regions resulting from inevitable localized short circuits in these electrodes, the specific capacitance continues to decrease with the increase in mass loading. In addition, about 91.9%, 93.6%, 95.1%, and 96.6% of their initial capacitance were remained for these electrodes with mass loading of 1.2, 6.2, 12.1 and 18.5 mg cm^−2^, respectively, indicating a high cycling stability. This can be ascribed to the ultrathin feature of birnessite films generating a flexible feature, which buffers the electrochemical stress during the charge–discharge process. Thus, no obvious cracks appeared on the electrode, and the material morphology remained unchanged after 10 000 cycles (Figure S20a,b, Supporting Information). In addition, an obvious enhancement in cycling stability is observed with increasing mass loading, ascribed to the release of structural stress through compact coating induced by electrochemical reactions. In detail, for high‐mass‐loading electrode, the mutual constraint between particles can disperse the stress and maintain the stability of the layered structure. In addition, with increasing mass loading, the conductive network maintains partial connectivity despite particle fragmentation (Figure S20c, Supporting Information). These results demonstrate the superiority of high‐mass‐loading electrodes, which are capable of meeting the demands of practical applications.

**Figure 4:**
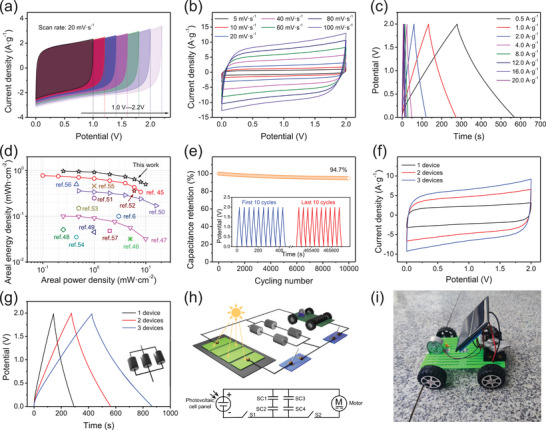
Electrochemical performance of SPB/CCB/CNTs// HPC/CCB/CNTs asymmetric device (electrolyte: 1.0 m Na_2_SO_4_): a) CV curves (potential window: 1.0‐2.2 V; scan rate:20 mV s^−1^), b) CV curves, c) GCD curves, d) Ragone plots (areal), e) cycling stability (insert: first and last 10 GCD cycles), f) CV curves (20 mV s^−1^) and g) GCD curves (1.0 A g^−1^) of SPB//HPC asymmetric devices (1–3) in “shunt” model, h,i) PV‐SC system.

Based on the favorable capacitive properties of SPB, high electrical conducting routes (CCB and CNTs) and optimal slurry concentration, high‐mass‐loading supercapacitors were assembled by SPB /CCB/CNTs electrode and HPC /CCB/CNTs electrode. By designing the porosity and adjusting the pore distribution, the HPC has displayed remarkable superiority in electrochemical performance including capacitance, rate performance, and cycling performance (Figure S21, Supporting Information). Moreover, the dead mass in HPC has been reduced significantly by rational pore design. Therefore, HPC is highly suitable for being assembled with SPB into high‐mass‐loading electrodes. The capacitance of HPC was calculated to be 232 F g^−1^ (1.0 A g^−1^) with the mass loading of 21.2 mg cm^−2^. Thus, the mass ratio of SPB and HPC was controlled to be 16.8 and 21.2 mg cm^−2^ with superfluous mass being ignored (Figure S22, Supporting Information). A wide operating window from 1.0 to 2.2 V were measured, indicating a high energy density of the asymmetrical device (**Figure** **4**a). The observed rectangular CV shapes for all curves indicate an ideal capacitive property of the device, benefited from the cooperation of positive electrode and negative electrode. Detailly, SPB electrode storage energy by Faradic pseudo‐capacitive reaction principally, which is an ultrafast kinetic process. While, the charges were stored by EDLC reaction for HPC electrode with an ultrafast surface adsorption‐desorption process. The rectangular CV shapes can be maintained even increasing the scan rate to 100 mV s^−1^, implying an ultrafast electrochemical response (Figure 4b). based on the nearly isosceles triangular GCD curves, a high capacitance of 71.2 F g^−1^ was achieved, indicating an ideal capacitive reaction process. This is ascribed to the construction of high‐speed ion transport pathways by CCB and CNTs in the positive/negative electrodes. Specifically, charges resulting from the energy storage reaction between the electrolyte and SPB/HPC are capable of rapid transfer to the current collector, resulting in a fast charge separation process. Hence, approximately 50% of the capacitance can be retained after increasing the current density to 20 A g^−1^, indicating a favorable rate performance of the device (Figure S23a, Supporting Information). Additionally, the areal capacitance of 1.75 F cm^−2^ indicates the ideal areal electrochemical properties, meeting the demand of commercial energy storage device. Hence, a maximum areal energy density of 0.97 mWh cm^−2^ was obtained (Figure 4d), based on the following equation:

(4) $$E_{a}=0.5\times C_{a}\times V^{2}/3.6$$

where *E*_a_ is the areal energy density, *C*_a_ is the areal capacitance, and *V* is the voltage window. Note that, this is a record value obtained for MnO_2_ based high‐mass‐loading supercapacitors.^[^ ^6^, ^45^, ^46^, ^47^, ^48^, ^49^, ^50^, ^51^, ^52^, ^53^, ^54^, ^55^, ^56^, ^57^ ^]^ Detailed comparative information was listed in Table S3 (Supporting Information). Furthermore, A maximal gravimetric energy density of 39.6 Wh kg^−1^ was calculated, which is ascribed to the high gravimetric capacitance and wide operating windows (Figure S23b, Supporting Information). These favorite properties can be attributed to the ultrathin feature of SPB generating abundant electrochemical active sites, high‐speed electronic transmission routes established by CCB and CNTs and the synergistic role of SPB and HPC electrode. The cycling performance of the device conducted with GCD curves (4.0 A g^−1^) was displayed in Figure 4e. As shown clearly, the capacitance retention rate can reach 94.7% after 10 000 cycles, with the last 10 GCD curves remaining unchanged compared with the first 10 cycles (in inset of Figure 4e), attributed to the high electrochemical stability of SPB and HPC, as well as an optimal working window of the devices.

To further highlight the practical application prospects, several asymmetric devices were connected in a parallel configuration. As shown in Figure 4f,g, all CV curves exhibit ideal rectangular shapes and GCD curves display standard triangular shapes, indicating favorable electrochemical properties of the devices after being connected in a shunt configuration. In addition, the area of the CV curves and the discharge time of the GCD curves are almost directly proportional to the number of devices, demonstrating the high uniformity of the prepared devices. Thus, a PV‐SC system was constructed. As shown in Figure 4h, this system comprises a photovoltaic panel (5 V, 0.675 W), 4 asymmetric devices connected in a “Tandem‐Shunt” configuration supplying 4 V, and a motor vehicle (3 V) with detail system circuit shown insert of Figure 4h. After being charged in sunlight for 60 seconds, this mechanical vehicle traveled a distance of 4.8 m compared to 0.3 m of commercial porous carbon‐based PV‐SC system, highlighting the practical application prospects of SPB‐based high mass‐loading electrodes (Figure 4i). These results highlight the superiority of SPB‐based high‐mass‐loading electrodes in practical applications, meeting the demand of high‐efficiency energy storage systems.

## 3 Conclusion

In summary, this work developed an F^−^‐regulated strategy to fabricate dense‐core‐free SPB nanostructures. F^−^ selectively adsorbs on the (001) plane of birnessite to suppress its growth, enabling precise control of nanosheet thickness to ≈3 nm. This dense‐core‐free structure with ultrathin films eliminates electrolyte‐inaccessible dense regions and exposes abundant MnO_6_ octahedral active sites, collectively enhancing electrochemical performance. Leveraging this structural advantage, high‐mass‐loading SPB electrodes (15.3–24.7 mg cm^−2^) were achieved by optimizing slurry concentration to minimize closed pores and constructing a CCB/CNTs 3D conductive network to alleviate electron transport bottlenecks. These electrodes exhibit reduced ohmic resistance and enhanced cycling stability, supporting the assembly of asymmetric supercapacitors with a record areal energy density of 0.97 mWh cm^−2^. These results provide a new insight for designing high‐performance energy storage electrodes by coupling structural regulation and conducting network optimization. Future studies will integrate continuous coating technology to enable scalable fabrication and tune pore distribution for better ion diffusion, further promoting the integration of SPB‐based electrodes into practical systems such as power vehicles, smart homes, and aerospace energy devices.

## Conflict of Interest

The authors declare no conflict of interest.

## Author Contributions

The manuscript was written through the contributions of all authors. All authors have approved the final version of the manuscript.

## Supporting information



Supporting Information

## Data Availability

The data that support the findings of this study are available from the corresponding author upon reasonable request.;
