# Supplementary material for: Boosting Electrochemical Capacitive of Ultrathin Shower‐Pouf Birnessite‐Type MnO2 for Porous High‐Mass‐Loading Energy Storage Device
Source: Adv Sci (Weinh). 2025 Oct 5;12(48):e13473. doi: 10.1002/advs.202513473 (PMC12752614; doi:10.1002/advs.202513473)
Supplement: Supplementary file 1 — Supporting Information [file ADVS-12-e13473-s001.docx]

**Boosting Electrochemical Capacitive of Ultrathin Shower-pouf Birnessite-type MnO_2_ for Porous High-mass-loading Energy Storage Device**

Shijin Zhu*, Chenxin Zhang, Minghao Sun, Fangdie He, Yuxin Liu, Xinyi Guo,

Mengwei Yao, Meng Zhao

*School of Materials and Chemical Engineering, Chuzhou University, Chuzhou, 239000, China*

*Corresponding author, E-mail addresses: zhushijin1112@chzu.edu.cn (Dr. S. Zhu)

**Experimental Section**

**Chemicals:** Potassium permanganate (KMnO_4_, ≥99.5%, Sinopharm), ammonium fluoride (NH_4_F, 99.7%, Xihua), polyacrylonitrile (PAN, average M_w_ 150,000), methanol (99.8%, Fluka), N, N-Dimethylformamide (DMF, ≥99.5%, Acros), potassium hydroxide (KOH, ≥85%, Merck), sodium sulfate (Na_2_SO_4_, 99.0%, Aladdin), potassium chloride (KCl, 99.8%, Shanghai Chemical Reagent Co. Ltd.), agar powder (95.5%, Sigma-Aldrich), poly(vinylidene fluoride) (PVDF, ≥99%, Mw~534 000 g·mol^-1^, Aladdin), N-methyl pyrrolidone (NMP, 99.9%, Aladdin), ethanol (99.5%, Aladdin), conductive carbon black (CCB, super P, Aladdin), nano-CaCO_3_ (9-40 nm, 97.5%, Acros).

***Preparation of Shower-pouf Birnessite (******SPB):*** Shower-pouf birnessite was synthesized via a modified solvothermal method. Typically, KMnO_4_ (0.02 M, 20 mL) and NH_4_F (0.02 M, 20 mL) were mixed together under stirring for 5 min. Immediately, the mixed solution was sealed in a 50 mL Teflon-lined stainless-steel autoclave and kept for 24 h at 120 ^o^C. After cooling to room temperature, the products were centrifuged and washed with DI water and ethanol alternately to remove the impurities. Finally, the solid products were collected and dried at 80 ^o^C to remove the residual solvent.

***Preparation of hierarchical porous carbon (HPC):*** Polyacrylonitrile (PAN, 6.0 g) was dissolved in 50 mL DMF, and then heated to 60 ^o^C under stirring until the solution became clear. 6.0 g nano-CaCO_3_ was also dispersed in 50 mL DMF under sonication for 30 min. Afterwards, these two solutions were mixed and stirred for 10 min at 60 ^o^C and sonicated for another 30 min. The uniform mixture was cast on the surface of a flat glass sheet by doctor blading and the resulting wet films transferred to a mixture solution with 90 % deionized water and 10 % v/v methanol, where they were maintained for 5 min. Then, the PAN-CaCO_3_ films were placed in DI water over night and finally dried at 60 ^o^C.

The prepared PAN-CaCO_3_ films were heated to 250 ^o^C at a rate of 4 ^o^C min^-1^ follow by an isothermal step of 45 min to allow cross-linking in the air. The cross-linked PAN films were carbonized at 1000 ^o^C for 2 h in a tube furnace under flowing argon at a rate of 4 ^o^C min^-1^ then cooled to room temperature naturally. Afterwards, the resulting powder was washed with 1.0 M HCl to remove residual salts followed by drying at 80 ^o^C overnight. Then, the washed carbon was mixed with KOH (powder) at a mass ratio of 1: 3 in 20 mL in deionized water forming a mixture solution and stirred overnight. Finally, the mixture solution was dried at 80 ^o^C over night to remove the extra water. The resulting powder was then heated to 800 ^o^C again for another 2 h in a tube furnace under flowing argon with ramp rate of 4 ^o^C min^-1^ for activation. Finally, the activated carbon was washed with 1.0 M HCl solution and DI water several times and dried in vacuum oven at 80 ^o^C overnight.

***Material Characterization:*** Scanning electron microscope (SEM, Sigma 300, Carl Zeiss, Germany) and transmission electron microscopy (TEM, FEI, Talos F200x, US) were used to characterize the morphologies and crystal information of birnessite. X-ray diffraction (XRD, Bruker D8 advance, Cu/Kα, Germany) was conducted to establish the crystallographic information. The specific surface area and pore size distributions were evaluated by N_2_ adsorption/desorption isotherm (Micromeritics, ASAP 2460, US). The chemical compositions were investigated by X-ray photoelectron spectroscopy (XPS, Thermo Scientific, K-Alpha, US) with a monochromatic Al Kα (hν = 1486.6 eV) X-ray source at 12 kV. The structure was investigated by Raman spectrometer (Horiba Lab, RAM HR Evolution, 100-4000 nm). The electrical conductivity was measured by Four-Point Probes (Lattice Electronics, ST2263, China).

***Density functional theory (DFT) calculation:*** All the spin calculations were performed in the framework of the density functional theory (DFT) with the projector augmented plane-wave method, as implemented in the Vienna ab initio simulation package (VASP). The generalzied gradient approximation proposed by Perdew, Burke, and Ernzerhof was selected for the exchange-correlation potential (GGA-PBE). The cut-off energy for plane wave was set to 400 eV. The energy criterion was set to 1×10^−6^ eV in iterative solution of the Kohn-Sham equation. In the DFT calculations, the (010) crystal plane of layered MnO_2_ (birnessite) was modeled alongside the (001), (100), and (110) planes for comparative analysis of F⁻ adsorption behavior. A vacuum thickness of 15 Å along the z-direction was applied to avoid periodic interactions. A Monkhorst-Pack mesh of 2×2×1 was used in K-sampling. In addition, the DFT+U correction was applied for Mn element, where U value is 3.9 eV. The adsorption energy of the adsorbates can be computed by the equation of

ΔE_ads_ = E_(surface+adsorbate)_-E_(surface)_-E_(adsorbate)_

where E_(surface+adsorbate)_, E_(surface)_, E_(adsorbate)_ are the total energy of surface with adsorbates, pure surface, free radicals, respectively.

***High Mass Loading Electrode Preparation:*** In a typical preparation process, SPB (158.8 mg, 79.4%), conductive carbon black (26 mg, 13%) and Carbon nanotubes (15.2 mg, 7.6%) were mixed in ethanol (50 mL) and under stirring for 10 min followed by ultrasonic for 15 min. The mixture was centrifuged (4500 r·min^-1^, 3 min) and dried at 80 ^o^C. Then, PVDF/NMP solution (50 mg·mL^-1^) was added into as-prepared mixture at a mass ratio of 90:10 (mass of mixture: 90%; mas of PVDF: 10%). Afterwards, the mixture was milled and removed to a small bottle with a certain amount of NMP added to forming slurry with the concentration of 20, 25, 30, 35, 40 and 45 wt%. This slurry was coated on the surface of a nickel foil by a doctor blade with the coating speed of 2 cm·s^-1^. Afterwards, the coated electrode was kept in a vacuum oven at 120 ^o^C for 12 h to remove the solvents and water. The mass loading on the electrode was calculated to be 1.2-33.6 mg·cm^-2^. Two electrodes were prepared for every testing project. If the measuring error exceeds 5%, this testing project needs to be repeated. The negative electrode was prepared using the same method by replacing birnessite with HPC.

To evaluate the mechanical strength, the prepared electrodes were pressed using a laboratory tablet press under a pressure of 30 MPa for 30 seconds. Then, a rubber bulb was used to blow off the detached fragments, followed by weighing on an analytical balance.

***Electrochemical Characterization:*** The electrochemical properties of the prepared electrode and asymmetric supercapacitor were conducted on electrochemical workstations (CHI 660E) in 1.0 M Na_2_SO_4_ electrolyte. In a three-electrode configuration, the SPB electrode and a platinum sheet electrode were used as working electrode and counter electrode, respectively. A saturated calomel electrode was used as reference electrode. The asymmetric supercapacitor was assembled by SPB as positive electrode, HPC as negative electrode and a Whatman filter paper as a separator.

Cycle voltammetry (CV), galvanostatic charge-discharge (GCD) and electrochemical impedance spectroscopy (EIS) experiments were conducted to evaluate the electrochemical properties of the electrodes and asymmetric supercapacitor. The CV curves were carried out at the scan rate ranging from 2 to100 mV s^−1^. The applied current in GCD curves was calculated based on the total mass of SPB or HPC, CCB and CNTs. EIS measurement was enforced by applying an alternating voltage (5 mV, 0.01 Hz to 100 kHz) at the open circuit potential. The specific capacitance calculating equations are listed as follows:

$$C_{1}=\frac{\int_{V_{0}}^{V_{0}+\Delta V} Iⅆ\nu}{s\times m\times\Delta V}$$

$$C_{2}=\frac{I\times\Delta t}{m\times\Delta V}$$

where *I*, *ΔV*, *Δt*, *s* and *m* are the charge–discharge current (A), operating potential window(V), discharging time (s), scan rate (V·s^-1^) and mass loading (g), respectively.

The energy density and power density calculating equations are listed as follows:

$$E=\frac{1}{7.2}\times C\times U^{2}$$

$$P=\frac{3600\times E}{\Delta t}$$

where *C* is the capacitance (F·g^-1^ or F·cm^-2^) of the asymmetric device, *U* is its working potential window of the device, *Δt* is the discharging time (s), *E* is energy density (Wh·kg^-1^ or Wh·cm^-2^).

The dead mass calculating equations in electrode are listed as follows:

γ=$m_{AM}$/($m_{AM}+m_{DM}$) = C_o_/C_t_

where $m_{AM}$(g) is activated mass, $m_{DM}$ (g) is dead mass., C_o_ (F·g^-1^) is obtained specific capacitance calculated from CV or GCD curves and Ct is theoretical capacitance (F·g^-1^).

***PV-SC system:***

(1) Car size: 116*123 mm

(2) Car weight: 80 g

(2) Motor size: 24*24 mm

(3) Working voltage: 1-12v

(4) Solar panel: Polycrystalline silicon

(5) Maximum output voltage (Solar panel) :5 V

(6) Maximum power (Solar panel): 0.675 W

(7) Maximum power (Solar panel): 110mA


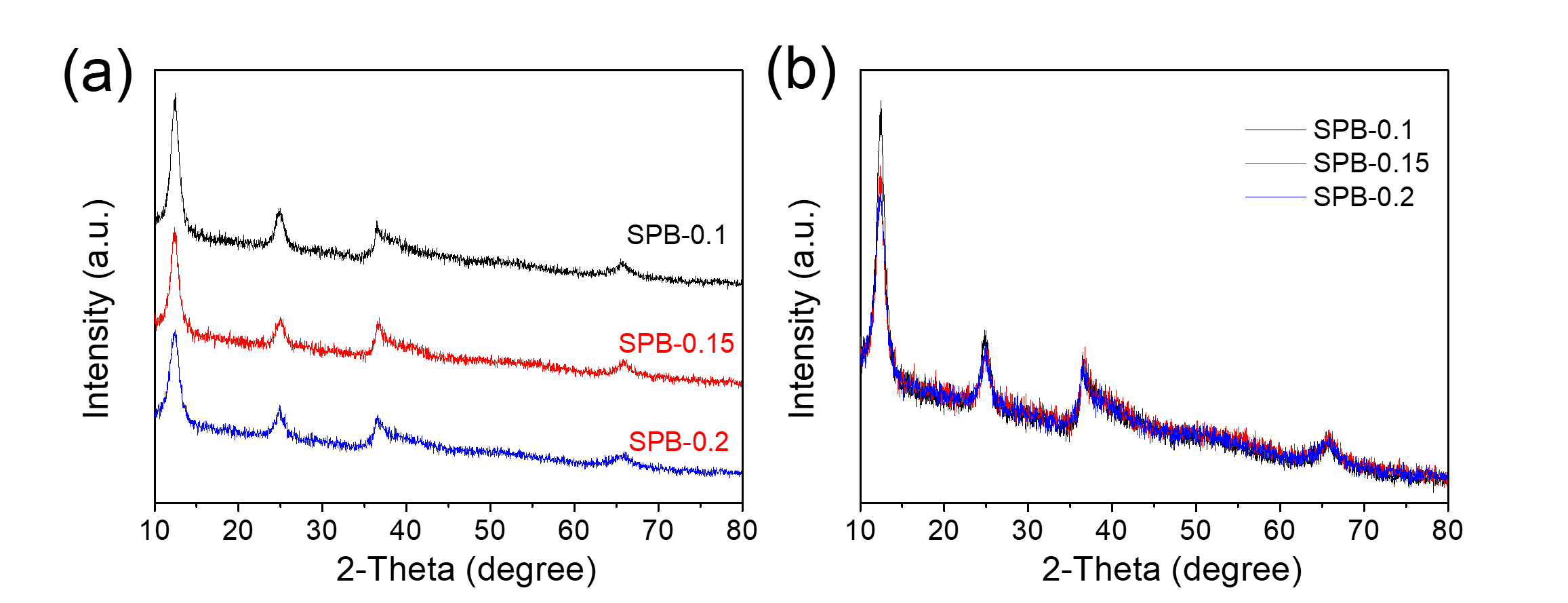


**Figure S1** (a, b) XRD pattern with different NH_4_F concentration (SPB-0.1: 0.01 M NH_4_F; SPB-0.15: 0.015 M NH_4_F; SPB-0.2: 0.02 M NH_4_F;)


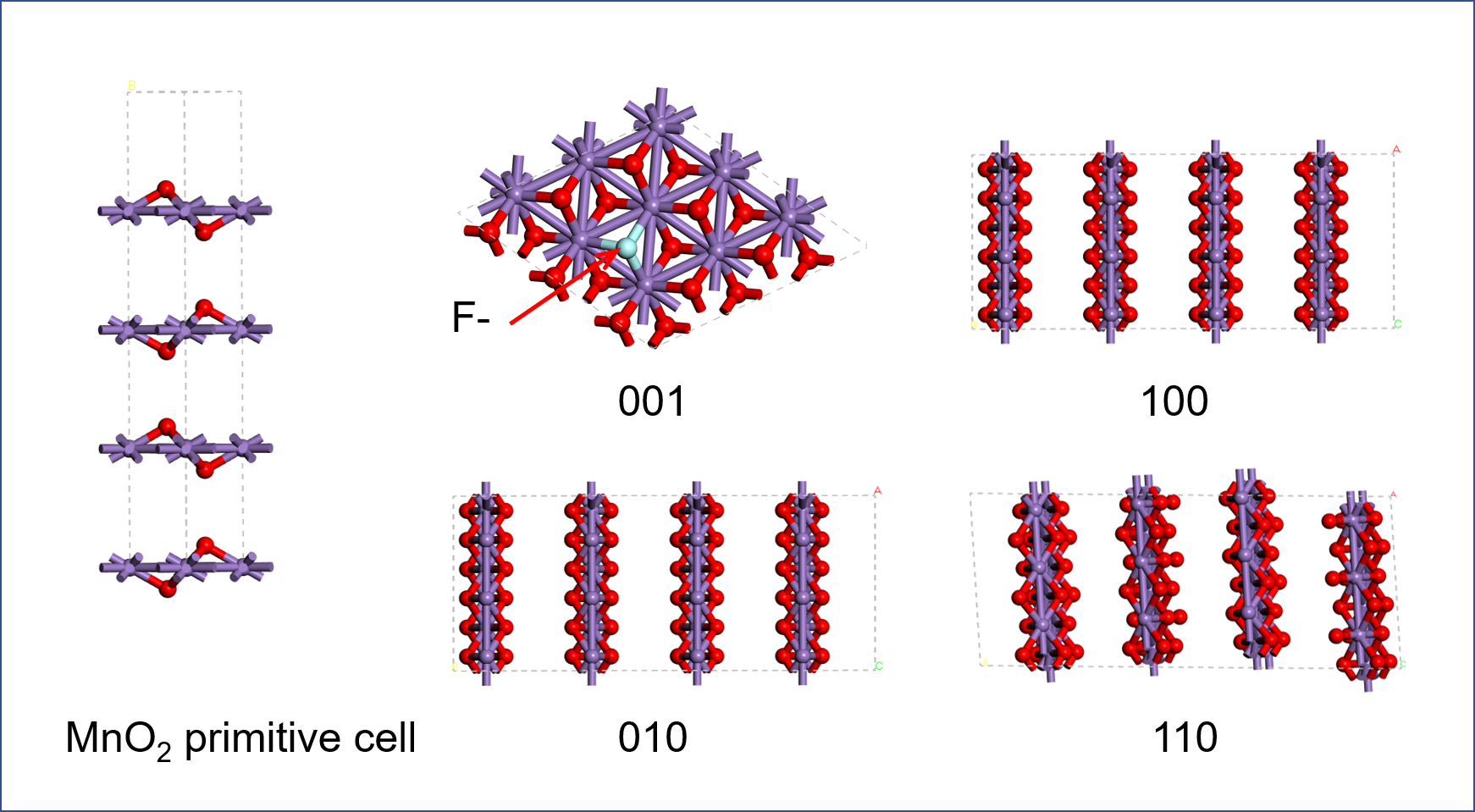


**Figure S2** TEM images of SPB nanostructures.


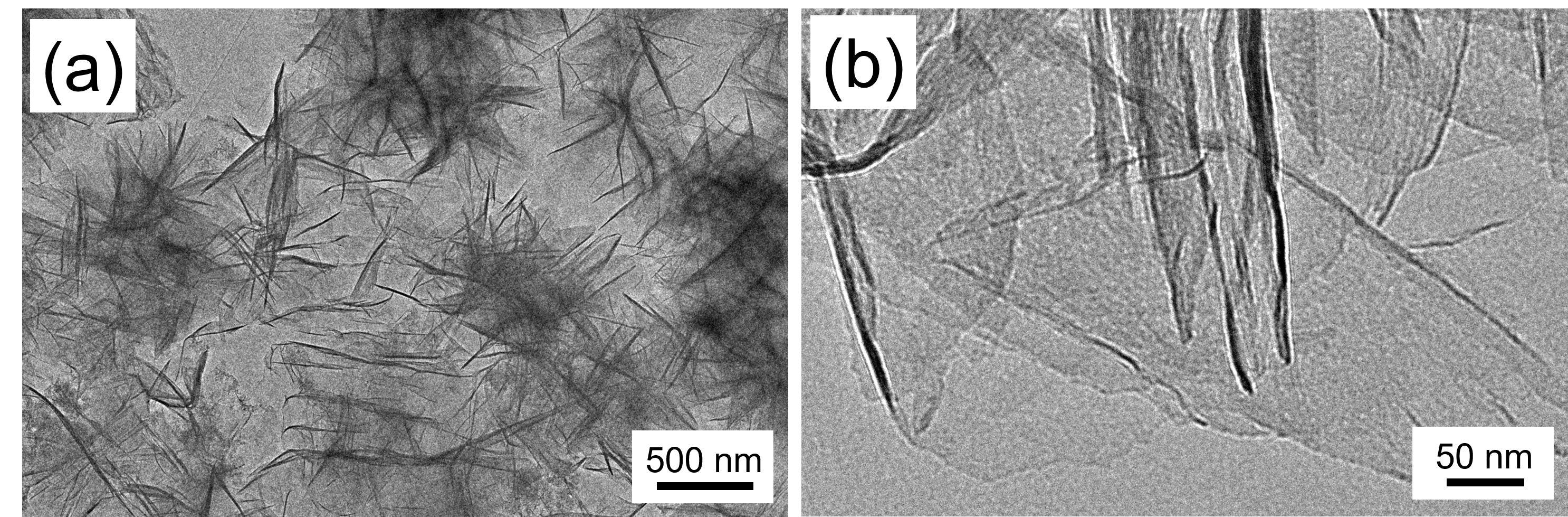


**Figure S3** TEM images of SPB nanostructures.


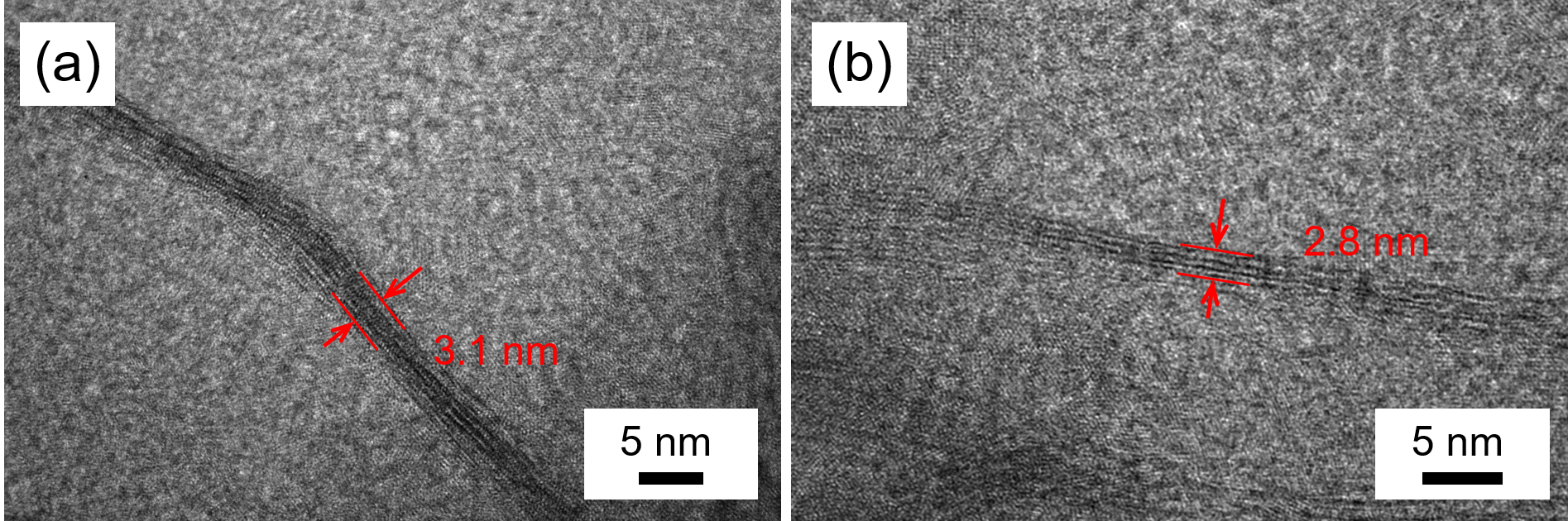


**Figure S4** HRTEM images of SPB nanostructures.


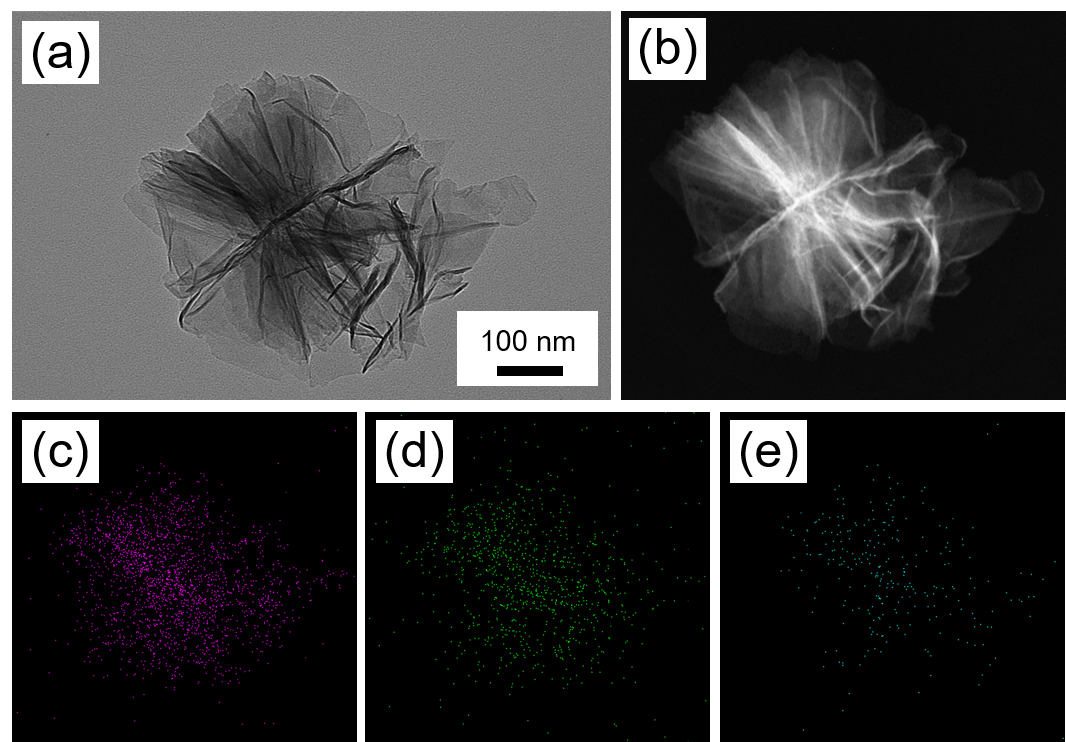


**Figure S5** (a)TEM images of SPB nanostructures. EDX mapping of SPB nanostructures: (b) morphology; (c) Mn; (d) O and (e) K.


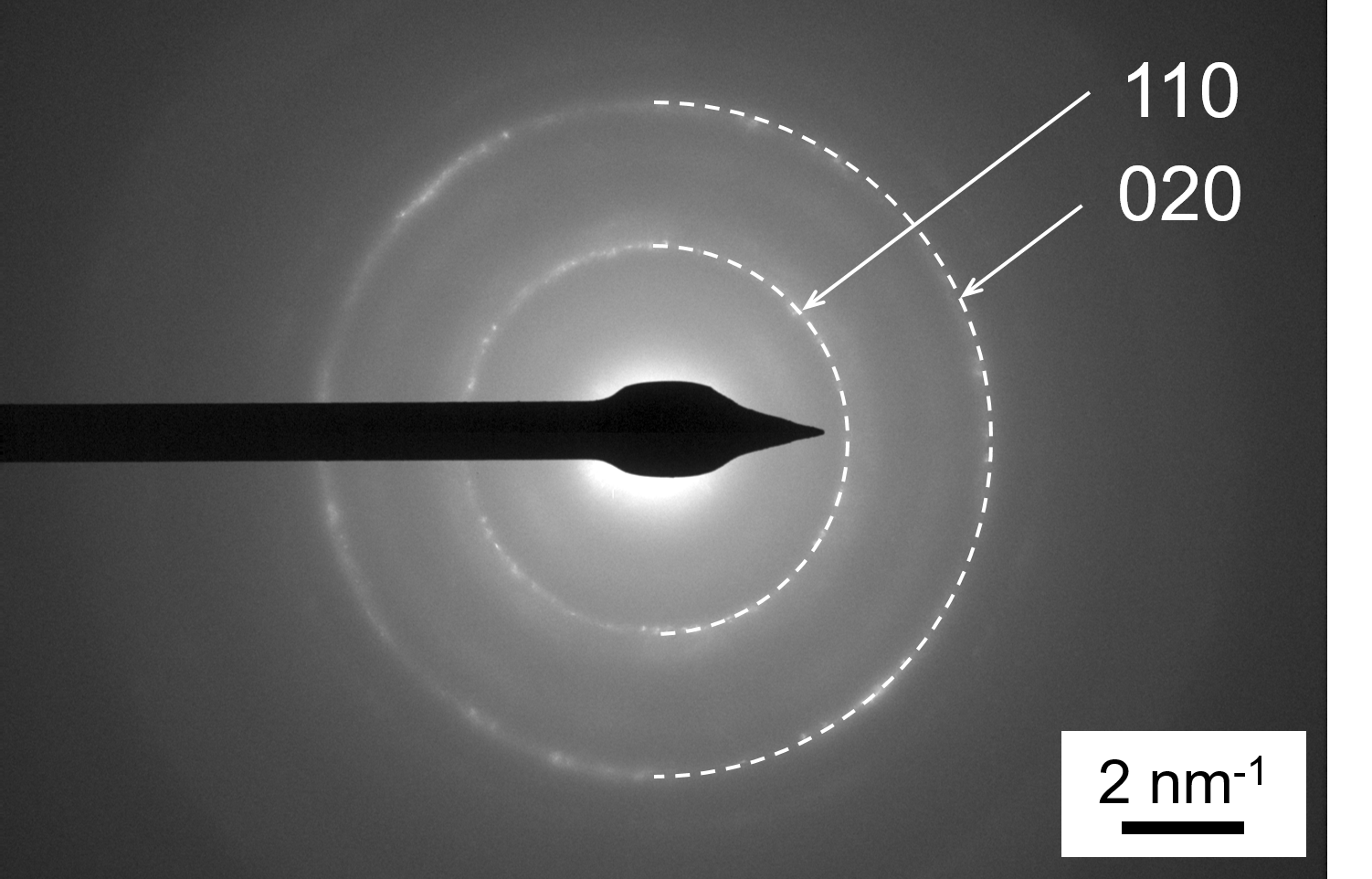


**Figure S6** Selected area electron diffraction of SPB nanostructures


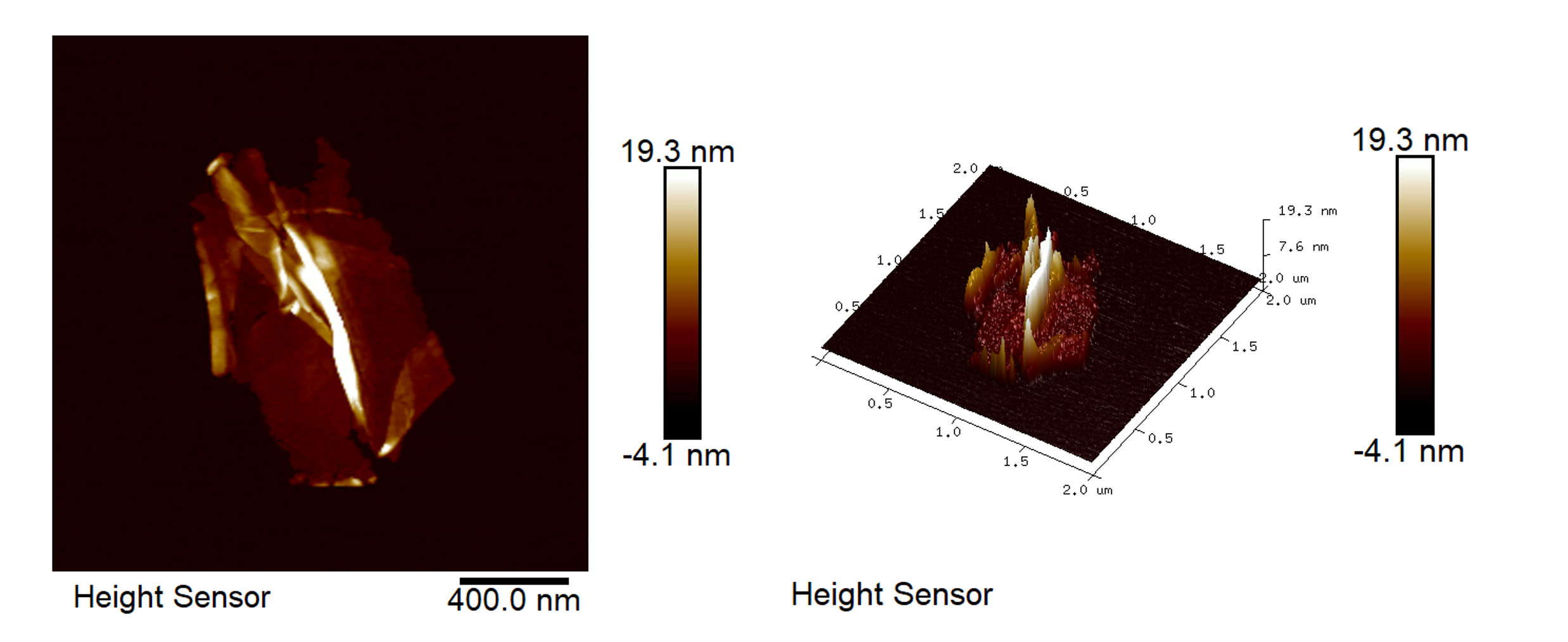


**Figure S7** (a)TEM images of SPB nanostructures. EDX mapping of SPB nanostructures: (b) morphology; (c) Mn; (d) O and (e) K.


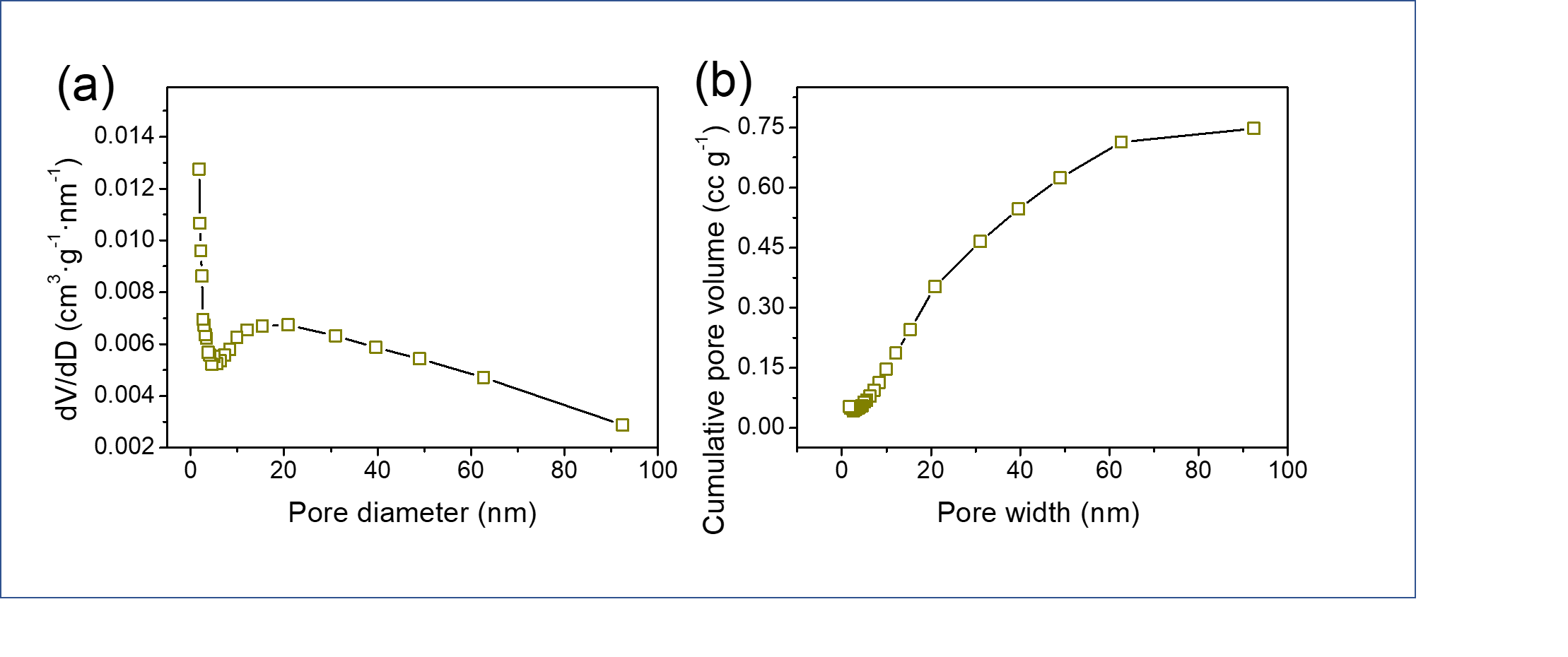


**Figure S8** Brunauer-Emmett-Tellern method of SPB: (a) Pore size distribution (b) cumulative pore volume.


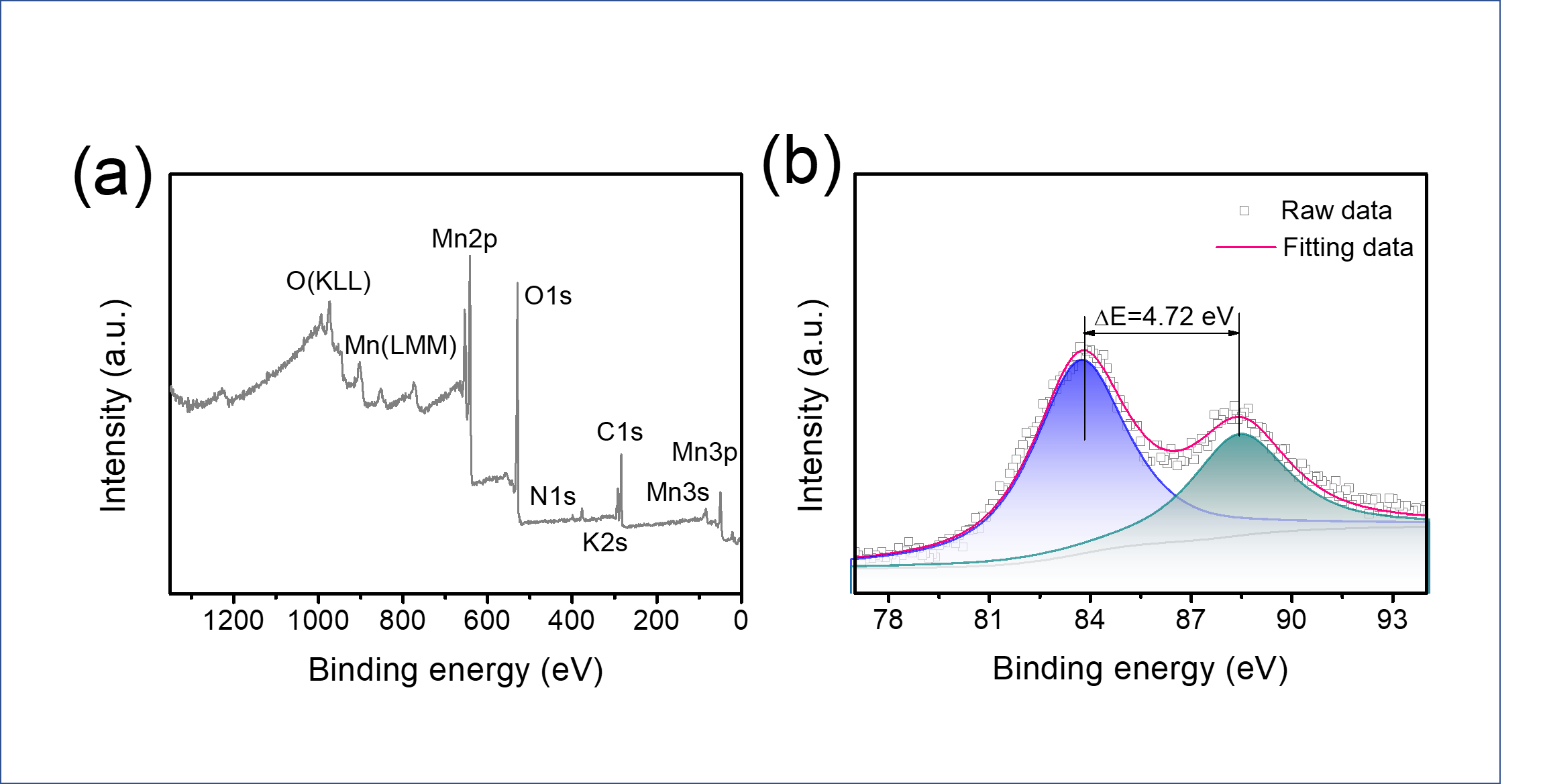


**Figure S9** Survey XPS spectrum of SPB nanostructure.


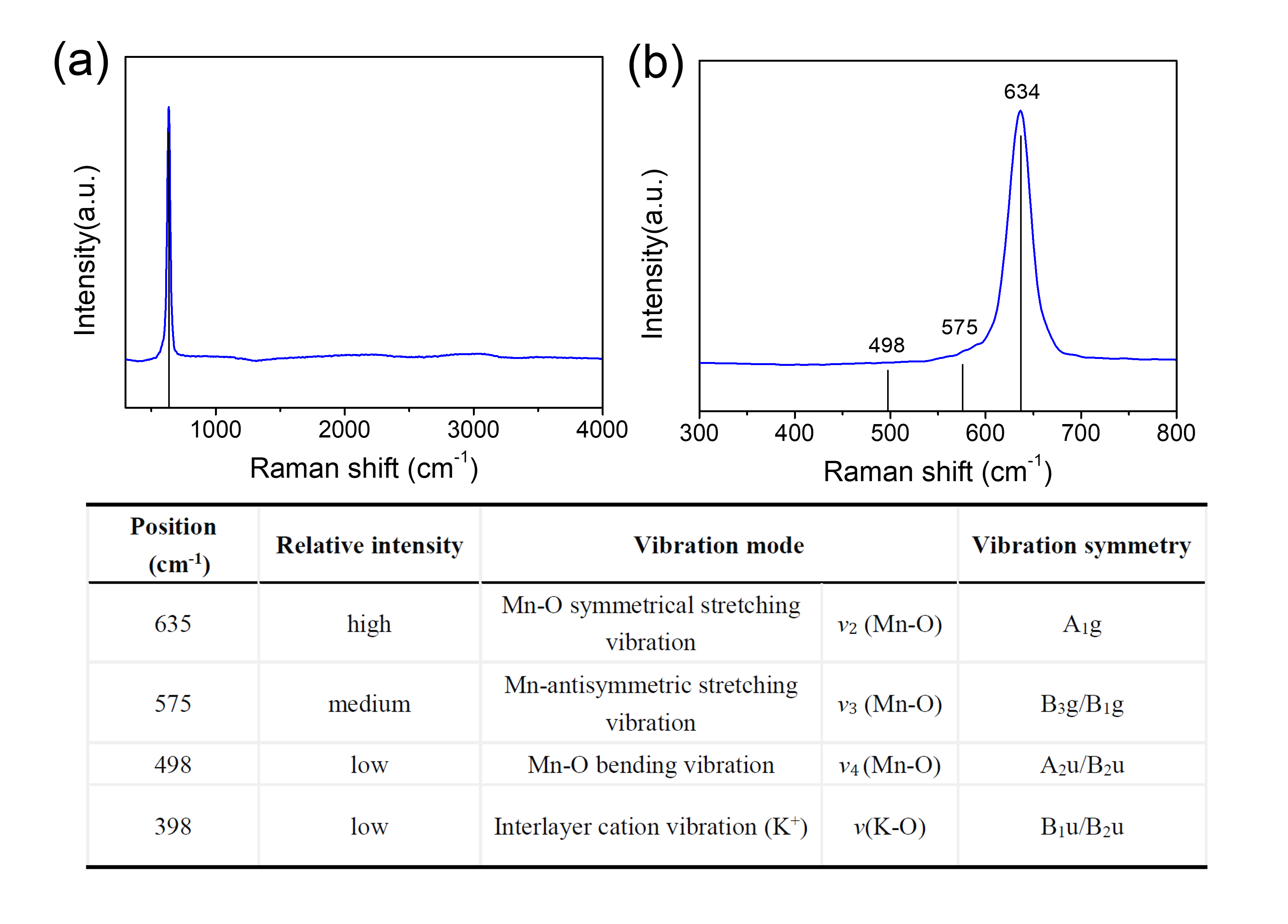


**Figure S10** Raman spectra of SPB nanostructure, (a) 300-4000 cm^-1^; (b)300-800 cm^-1^.

**
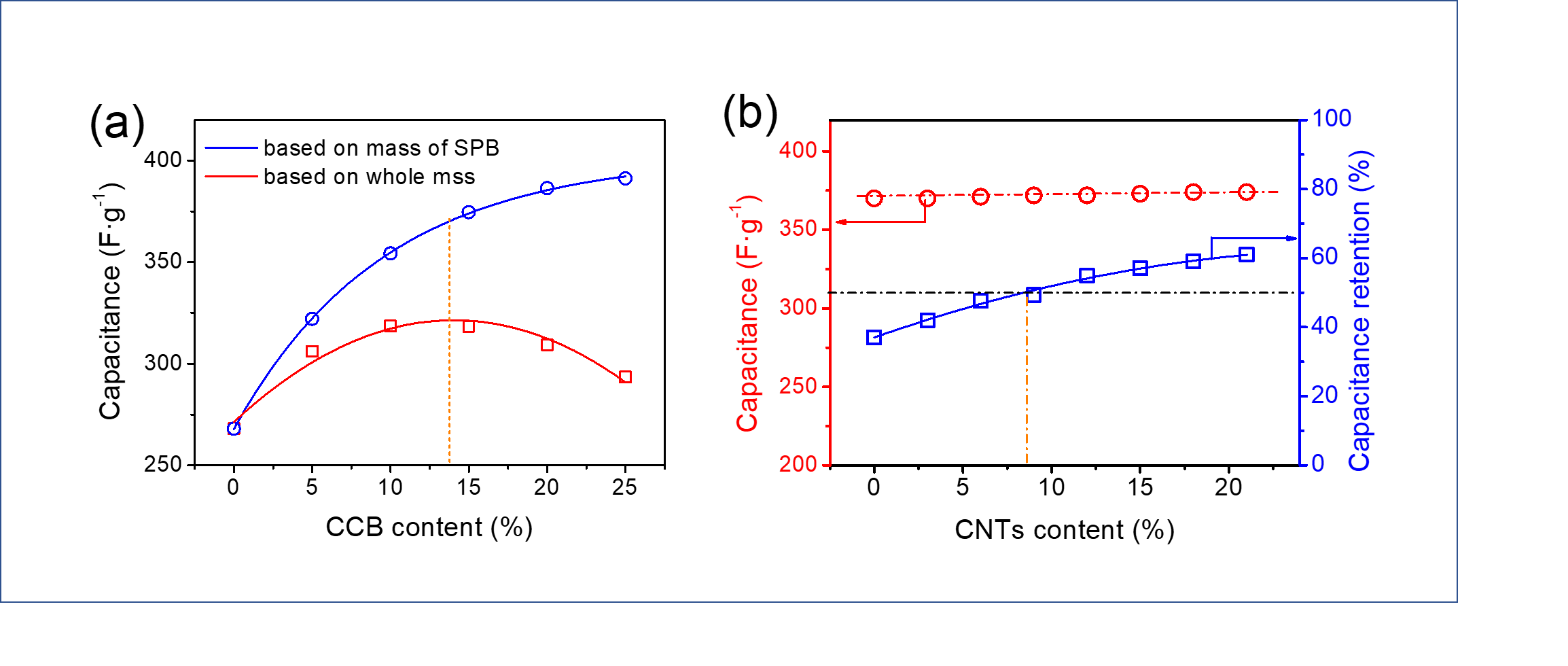
**

**Figure S11** (a) specific capacitance vibration of SPB with CCB content, (b) specific capacitance and capacitance retention of SPB with CNTs content.


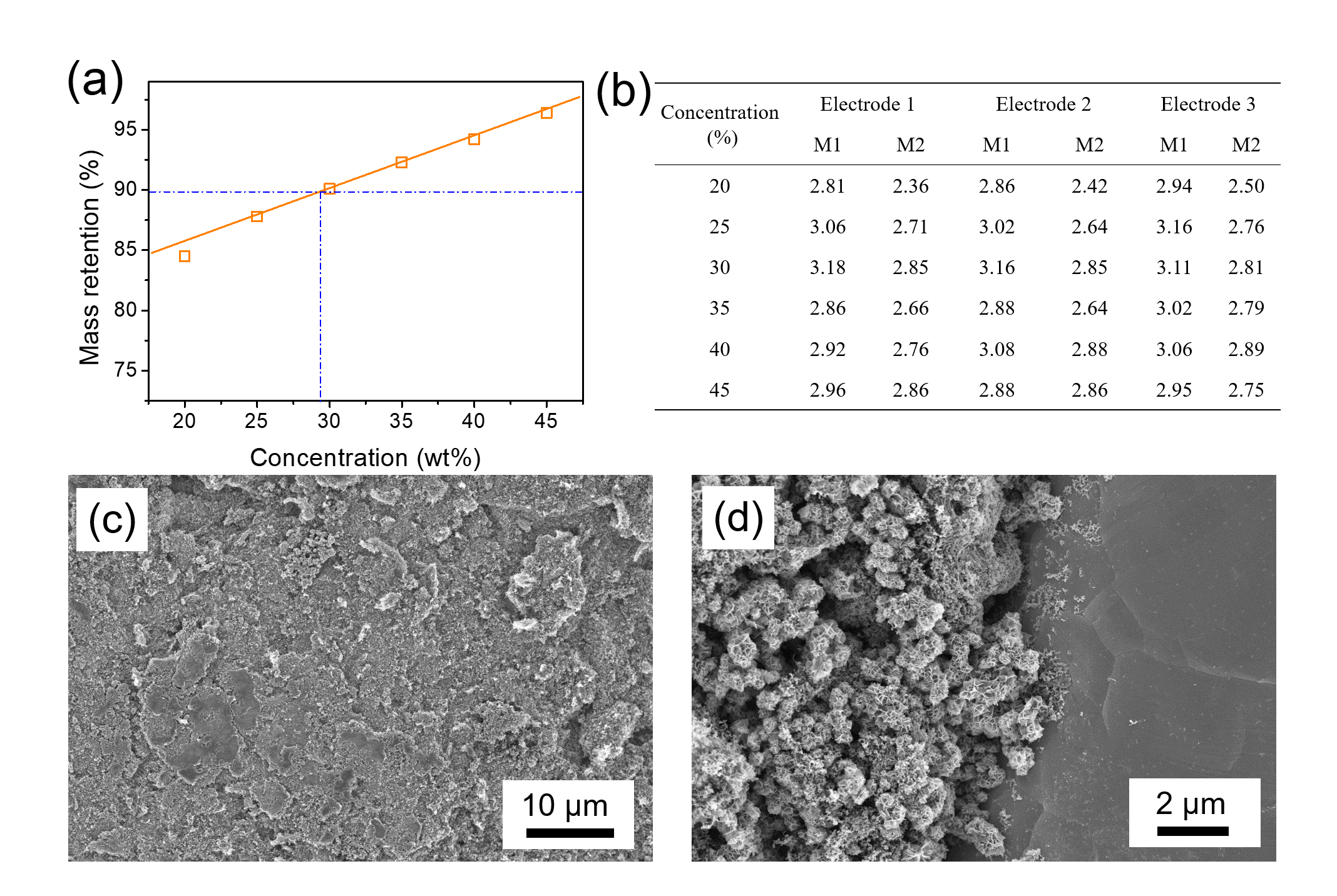


**Figure S12** (a, b) mass retention of active materials after being pressed under 30 MPa (M1: mass of electrode before being pressed, M2: mass of electrode after being pressed). (c, d) SEM images of SPB electrode after being pressed (30 wt%).

**Table S1** Electrochemical performance of MnO_2_-based high-mass-loading electrode.

| **NO.** | **Electrode** | **Mass loading**  **(mg cm^-2^)** | **Gravimetric capacitance**  **(F g^-1^)** | **Areal capacitance**  **(F cm^-2^)** | **Scan rate/**  **Current density** | **Electrolyte** | **Ref.** |
| --- | --- | --- | --- | --- | --- | --- | --- |
| 1 | MnO_2_/3D-Ni | 3 | 386.5 | 0.84 | 0.5 mA cm^-2^ | 0.5 M Na_2_SO_4_ | [1] |
| 2 | MnO_2_/polyaniline | 3 | 253 | 0.76 | 2 mV s^-1^ | 0.5 M Na_2_SO_4_ | [2] |
| 3 | GNF@NiCo_2_O4/MnO_2_ | 3.5 | unknown | 1.63 | 2.0 mA cm^-2^ | 1.0 M Na_2_SO_4_ | [3] |
| 4 | MnO_2_/graphite | 3.7 | 226 | 0.83 | 1.4 mA cm^-2^ | 1.0 M Na_2_SO_4_ | [4] |
| 5 | MnO_2_/TCC | 4.5 | 464 | 2.09 | 4.0 mA cm^-2^ | 5.0 M LiCl | [5] |
| 6 | MnO_2_ nanograins | 4.58 | unknown | 1.15 | 2.0 mA cm^-2^ | 1.0 M Na_2_SO_4_ | [6] |
| 7 | C@MnO_2_ | 6 | 177 | unknown | 0.5 mA cm^-2^ | 1.0 M Na_2_SO_4_ | [7] |
| 8 | MnO_2_-carbon | 6.5 | 230 | 1.5 | 2 mV s^-1^ | 1.0 M Na_2_SO_4_ | [8] |
| 9 | CDC@MnO_2_ | 6.6 | unknown | 1.1 | 1.0 mA cm^-2^ | PVA/LiClO_4_ | [9] |
| 10 | MnO_2_@CF | 6.6 | unknown | 1.19 | 2.0 mA cm^-2^ | 1.0 M Na_2_SO_4_ | [10] |
| 11 | MnO_2_/PANi | 8.3 | 423.7 | 3.52 | 5 mV s^-1^ | 1.0 M Na_2_SO_4_ | [11] |
| 12 | MnO_2_-CNT | 8.3 | 410 | 2.8 | 0.05 mV s^-1^ | 0.5 M Na_2_SO_4_ | [12] |
| 13 | CNT/MnO_2_/graphene | 9.1 | 371.4 | 3.38 | 1.0 mA cm^-2^ | 1.0 M Na_2_SO_4_ | [13] |
| 14 | graphene/MnO_2_ | 9.8 | 145 | 1.42 | 2.0 mV s^-1^ | 0.5 M Na_2_SO_4_ | [14] |
| 15 | MnO_2_ | 10 | 304 F | 3.04 | 3.0 mA cm^-2^ | 1.0 M Na_2_SO_4_ | [15] |
| 16 | 3D MnO_2/_PANI | 10.8 | 258 | unknown | 10 mV s^-1^ | 0.5 M Na_2_SO_4_ | [16] |
|  |  |  |  |  |  |  |  |
| **NO.** | **Electrode** | **Mass loading**  **(mg cm^-2^)** | **Gravimetric capacitance**  **(F g^-1^)** | **Areal capacitance**  **(F cm^-2^)** | **Scan rate/**  **Current density** | **Electrolyte** | **Ref.** |
| 17 | MnO_2_ | 12 | 443 | 5.32 | 2.0 mA cm^-2^ | 1.0 M Na_2_SO_4_ | [17] |
| 18 | MWCNT/ MnO_2_/rGO | 12 | 314.6 | unknown | 5 mV s^-1^ | 1.0 M Na_2_SO_4_ | [18] |
| 19 | WC@MnO_2_-20 | 14.1 | 45 | 1.56 | 2.0 mA cm^-2^ | 0.5 M Na_2_SO_4_ | [19] |
| **20** | **SPB/CCB/CNTs** | **15.3** | **278.6** | **4.26** | **2 mV s^-1^** | **1.0 M Na_2_SO_4_** | **This work** |
| 21 | Ag_2_O/H/MnO_2_ | 14.52 | 374 | 5.4 | 1.0 mA cm^-2^ | 1.0 M Na_2_SO_4_ | [20] |
| 22 | MnO_2_@CC | 15.1 | 213 | 3.2 | 1.0 mA cm^-2^ | 0.5 M Na_2_SO_4_ | [21] |
| 23 | Cellulose/f-CNT/ MnO_2_ | 15.93 | unknown | 7.95 | 1.0 mA cm^-2^ | 1.0 M Na_2_SO_4_ | [22] |
| 24 | alpha-MnO_2_/CC | 16 | 202.5 | 3.24 | 5.0 mA cm^-2^ | 1.0 M Na_2_SO_4_ | [23] |
| 25 | CNF/CNT/ MnO_2_ | 16.8 | unknown | 3.48 | 1.0 mA cm^-2^ | PAM/LiCl | [24] |
| 26 | MnO_2_/C | 19.7 | 480.3 | 9.4 | 0.5 mA cm^-2^ | 1.0 M Na_2_SO_4_ | [25] |
| **27** | **SPB/CCB/CNTs** | **24.7** | **203** | **5.01** | **2 mV s^-1^** | **1.0 M Na_2_SO_4_** | **This work** |
| 28 | MnO_2_/HGF | 28.2 | 260 | 7.35 | 1.0 mA cm^-2^ | 1.0 M Na_2_SO_4_ | [26] |
| 29 | MnO_2_/MWCNT | 30 | unknown | 4.4 | 2 mV s^-1^ | 0.5 M Na_2_SO_4_ | [27] |
| 30 | MnO_2_ | 30 | unknown | 4.75 | 1.0 mA cm^-2^ | 1.0 M Na_2_SO_4_ | [28] |
| 31 | MnO_2_/CNT | 47 | unknown | 7.52 | 2 mV s^-1^ | 0.5 M Na_2_SO_4_ | [29] |
| 32 | MnO_2_/CNT | 50 | unknown | 2.58 | 1.0 mA cm^-2^ | 1.0 M Na_2_SO_4_ | [30] |

**
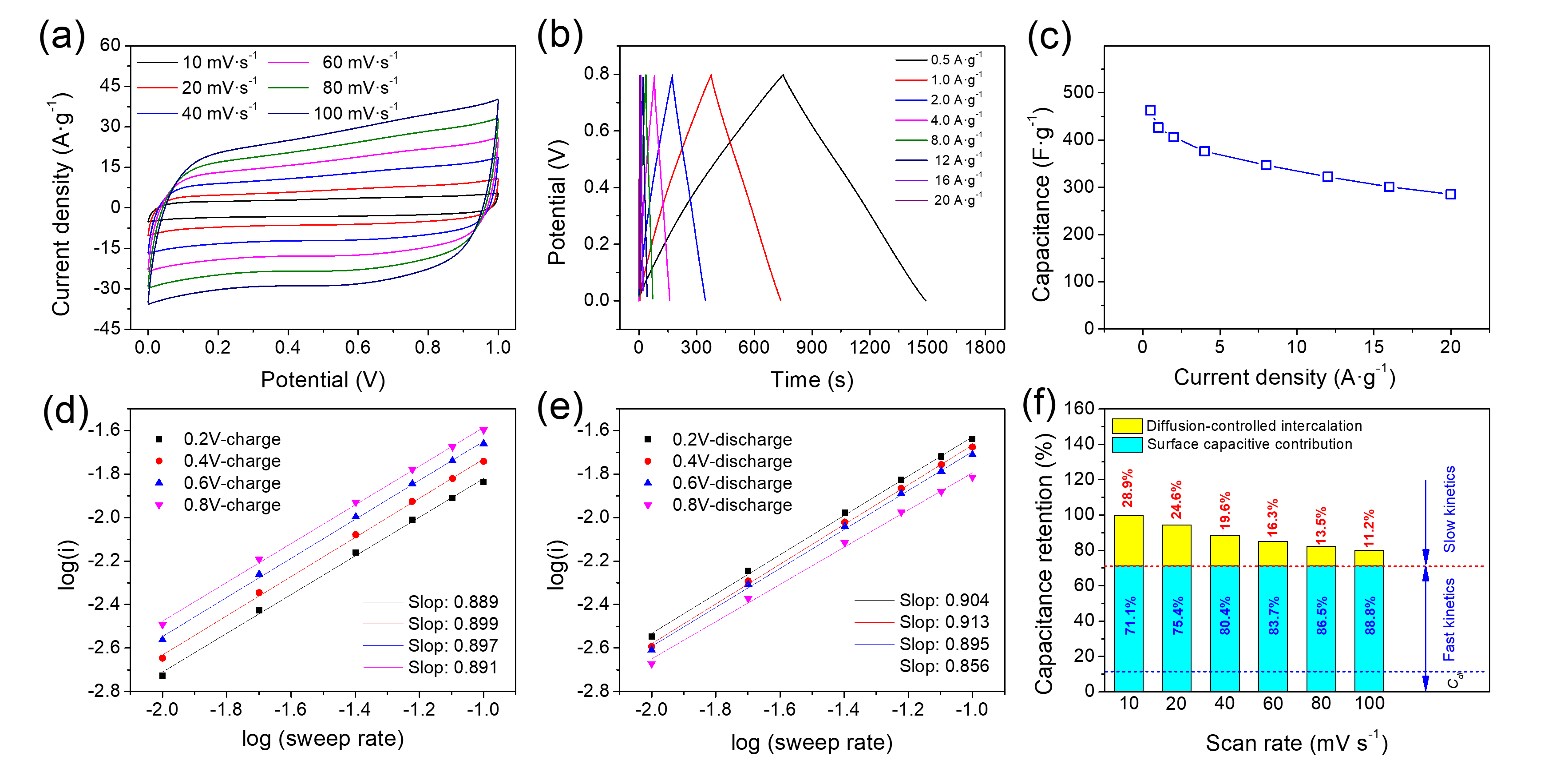
**

**Figure S13** Power law dependence of charge and discharge currents on various scan rates for SPB with mass loading of 1.2 mg·cm^-2^: (a) CV curves; (b) charge process; (c) discharge process.


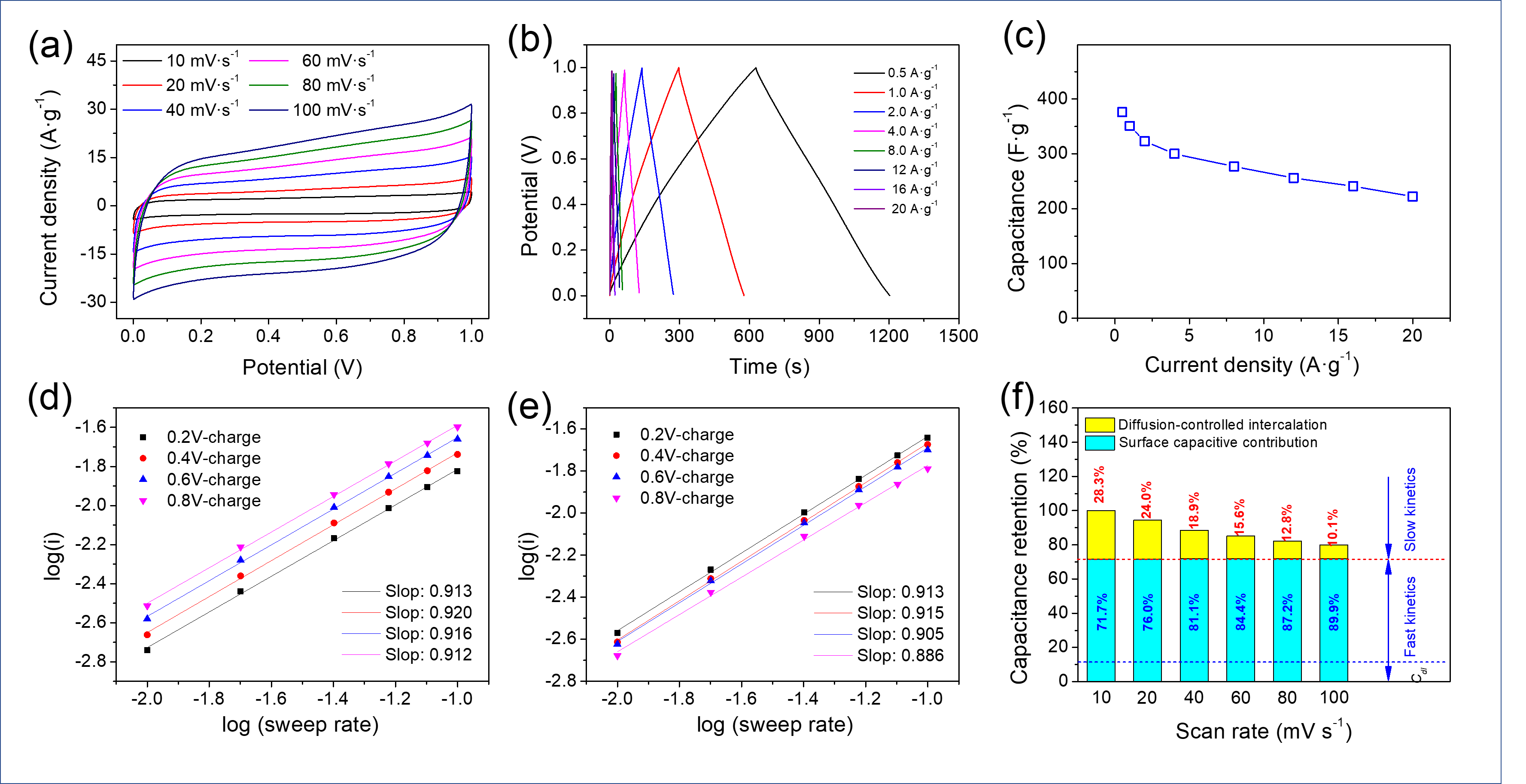


**Figure S14** Power law dependence of charge and discharge currents on various scan rates for SPB with mass loading of 3.1 mg·cm^-2^: (a) CV curves; (b) charge process; (c) discharge process.


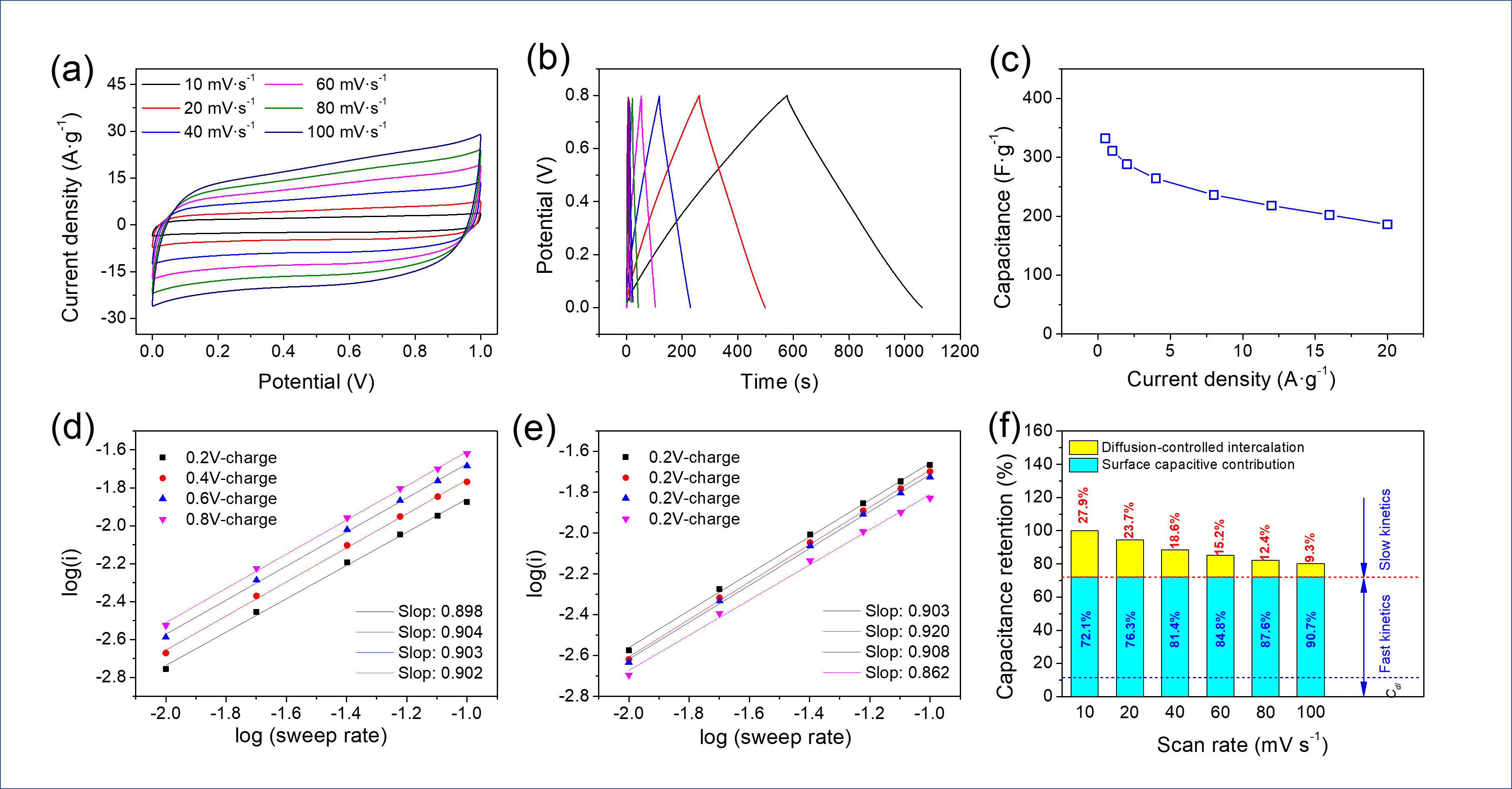


**Figure S15** Power law dependence of charge and discharge currents on various scan rates for SPB with mass loading of 6.2 mg·cm^-2^: (a) CV curves; (b) charge process; (c) discharge process.


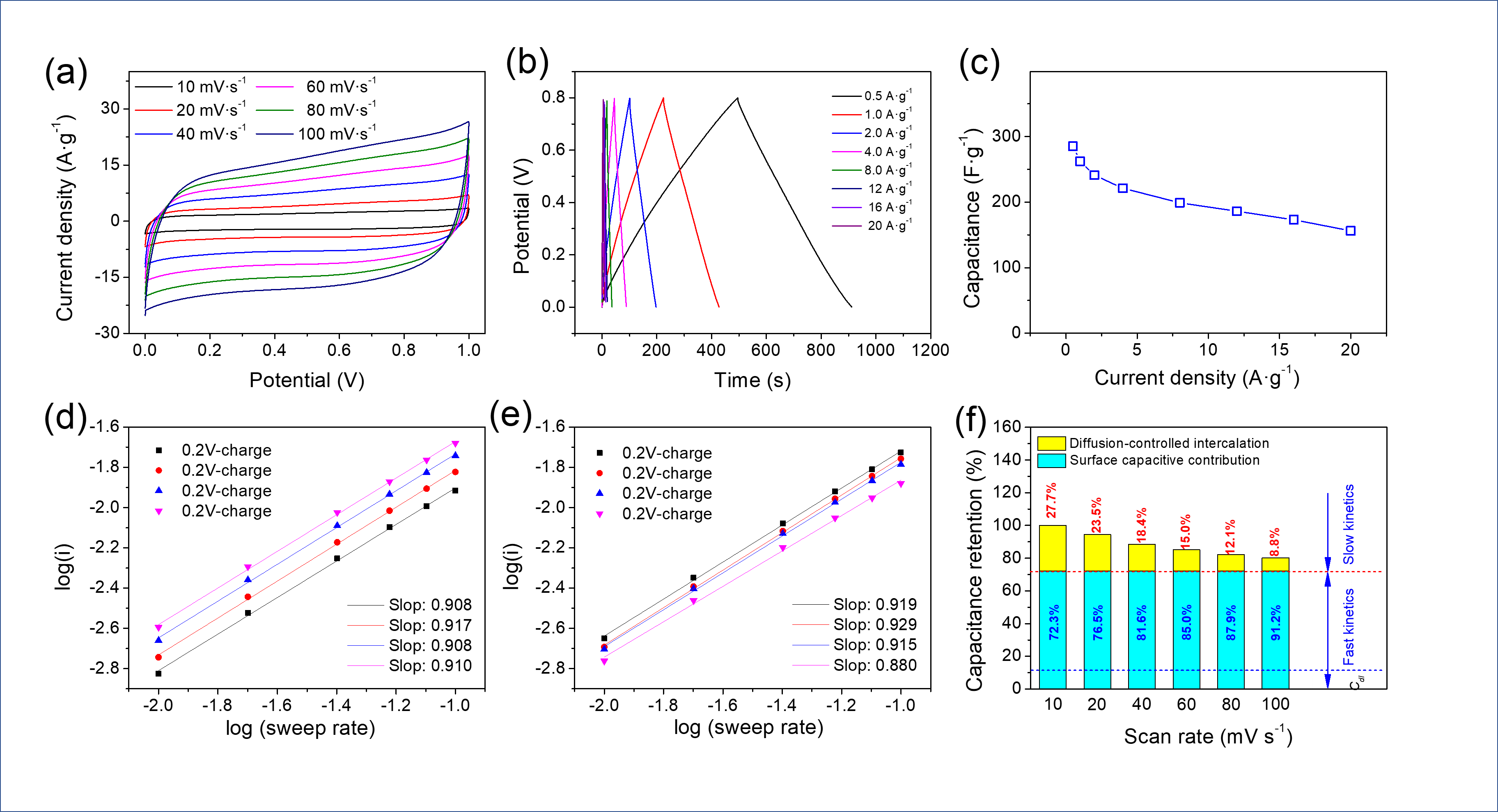


**Figure S16** Power law dependence of charge and discharge currents on various scan rates for SPB with mass loading of 12.1 mg·cm^-2^: (a) CV curves; (b) charge process; (c) discharge process.


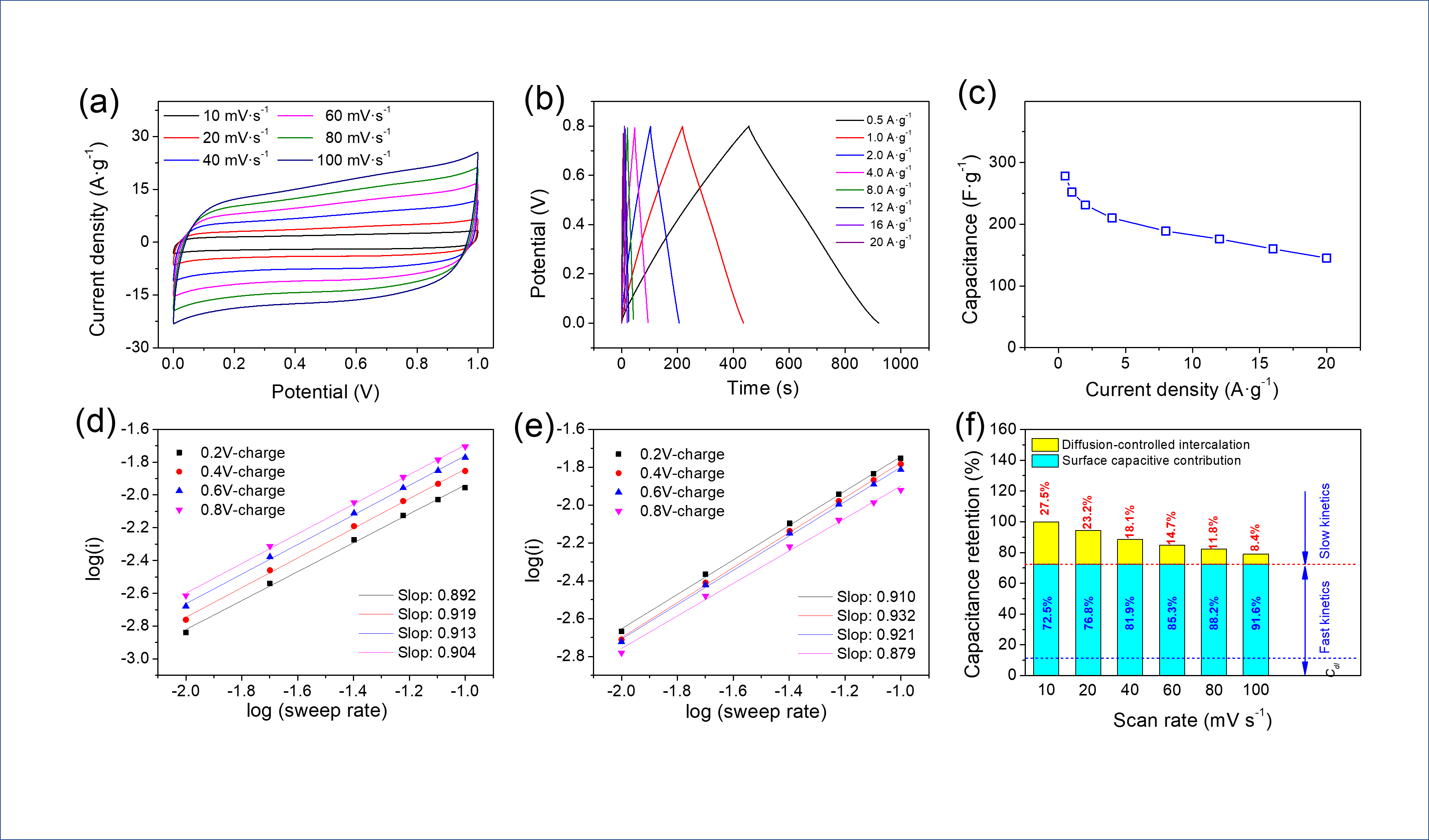


**Figure S17** Power law dependence of charge and discharge currents on various scan rates for SPB with mass loading of 15.3 mg·cm^-2^: (a) CV curves; (b) charge process; (c) discharge process.


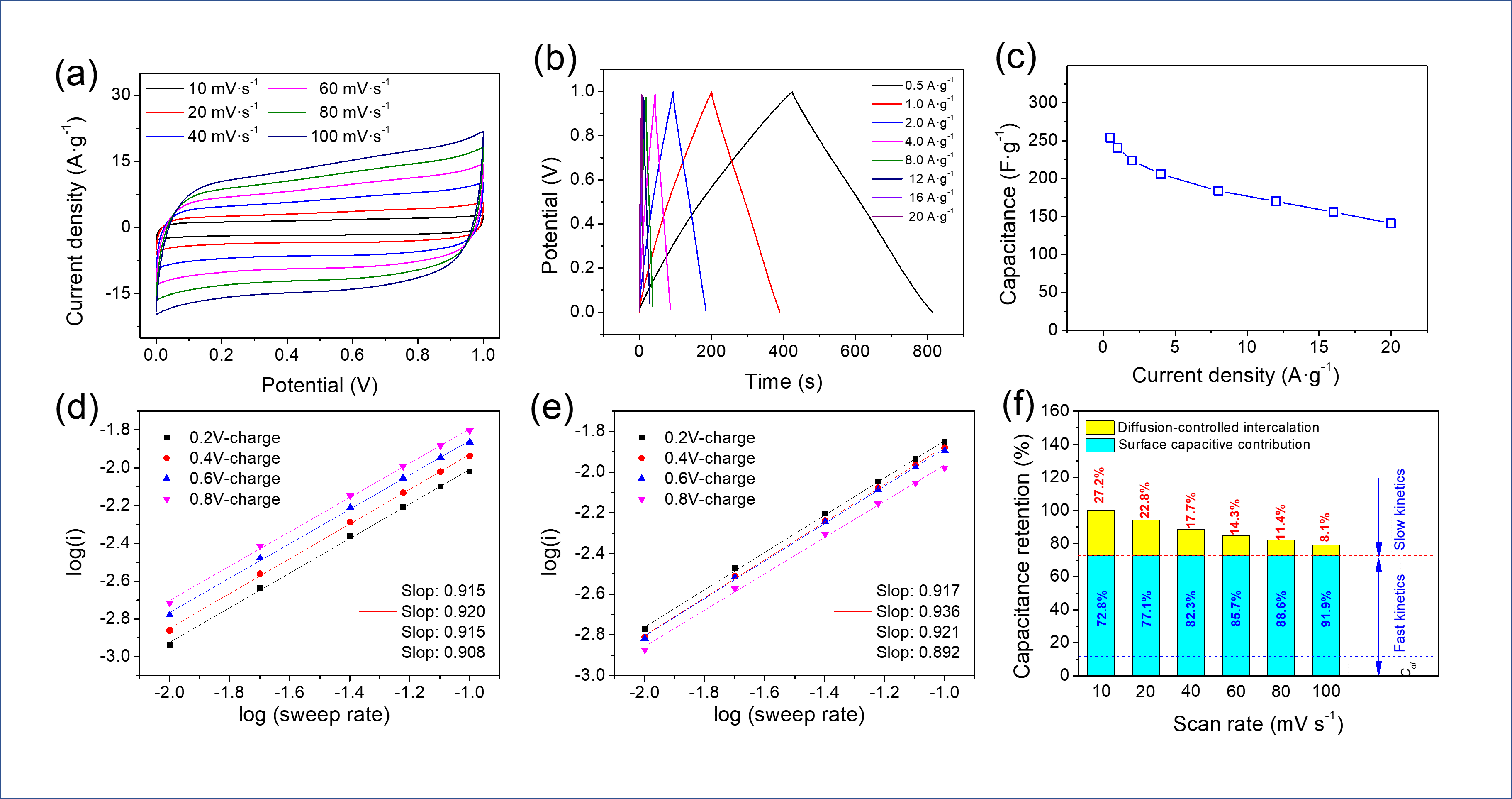


**Figure S18** Power law dependence of charge and discharge currents on various scan rates for SPB with mass loading of 18.5 mg·cm^-2^: (a) CV curves; (b) charge process; (c) discharge process.


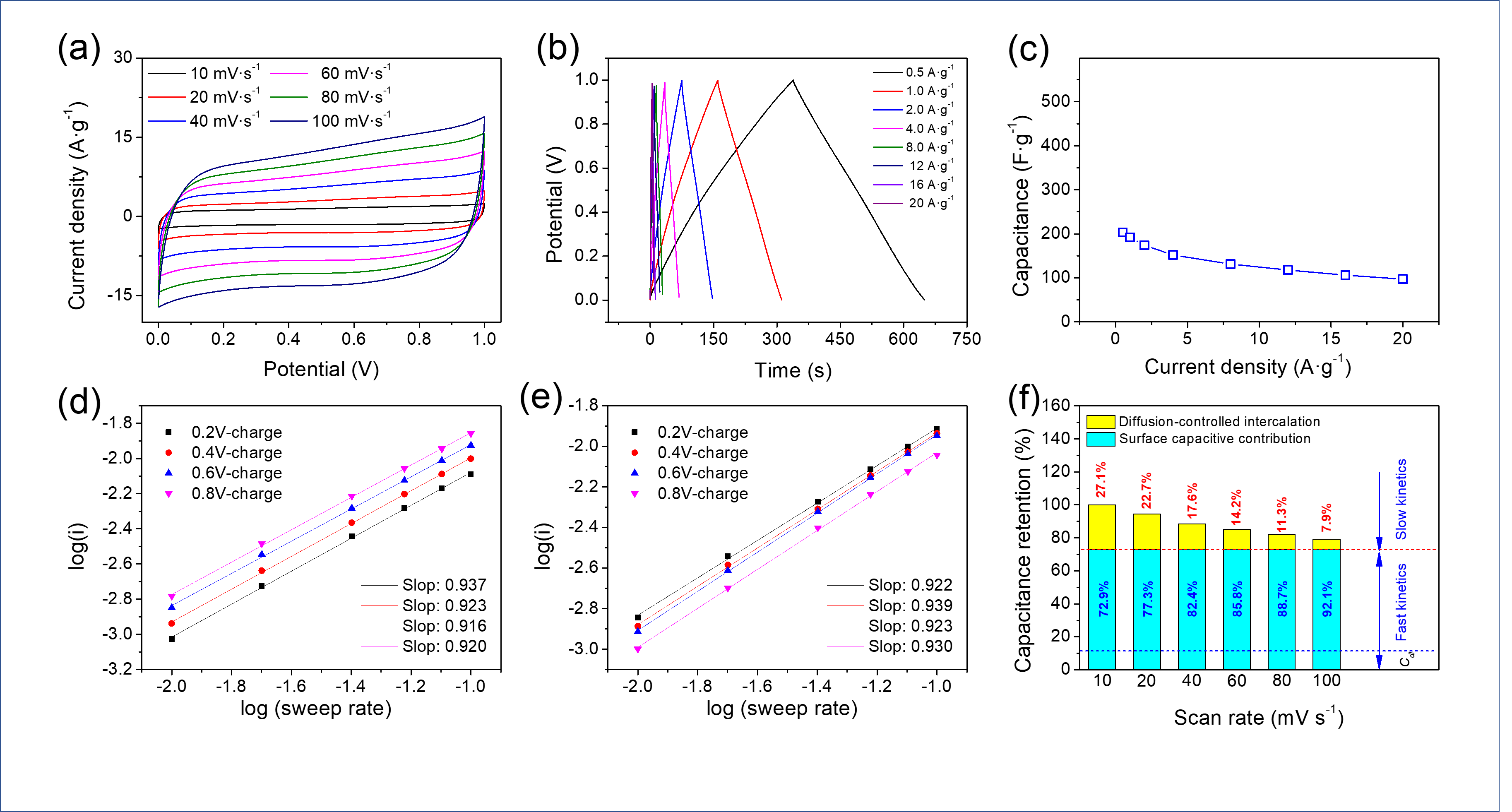


**Figure S19** Power law dependence of charge and discharge currents on various scan rates for SPB with mass loading of 24.7 mg·cm^-2^: (a) CV curves; (b) charge process; (c) discharge process.

| mass loading  (mg·cm^-2^) | R_s_  (Ω) | R_ct_  (Ω) | Z_w_  (s·sec5) | C_1_  (F) | C_2_  (F) |
| --- | --- | --- | --- | --- | --- |
| 1.2 | 2.178 | 1.463 | 0.122 | 3.181 | 0.4232 |
| 6.2 | 2.029 | 1.382 | 0.1825 | 0.0003 | 0.442 |
| 12.1 | 1.994 | 1.261 | 0.2189 | 0.395 | 0.3691 |
| 18.5 | 1.867 | 1.689 | 0.357 | 0.0002439 | 5.381 |

**Table S2** Calculated component values of equivalent circuit diagram for EIS spectrum.


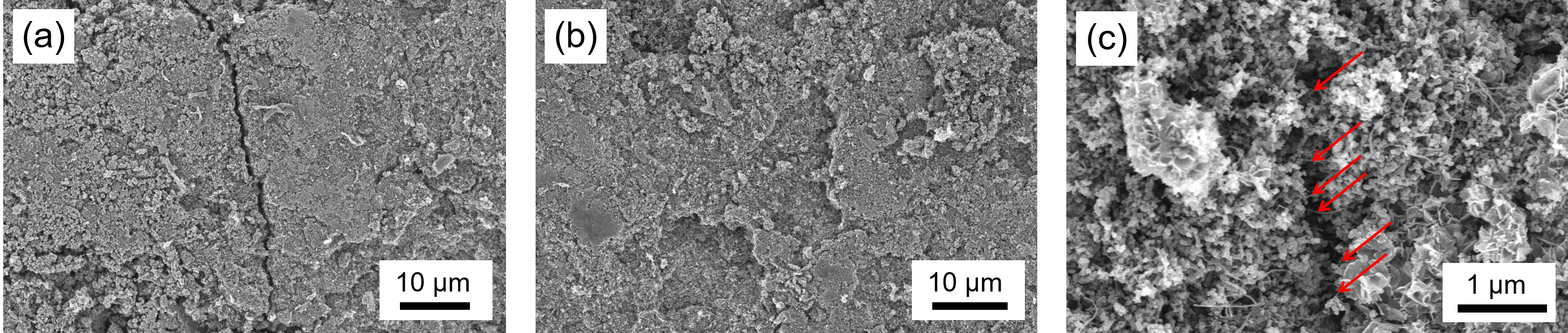


**Figure S20** SEM images of SPB electrode with mass loading 3.3 mg·cm^-2^ (a) and 14.6 mg·cm^-2^ (b,c) after 10000 cycles.


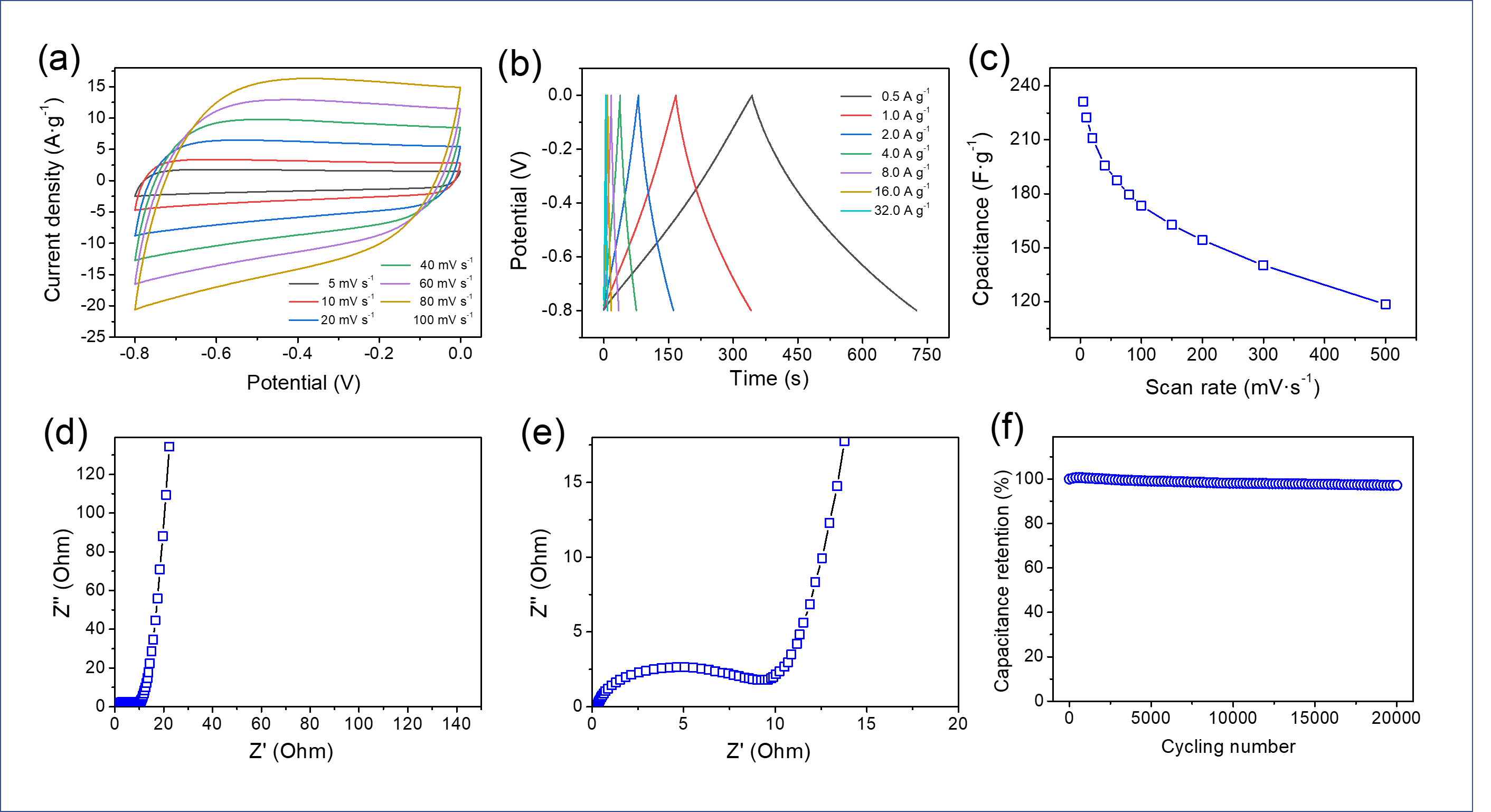


**Figure S21** Electrochemical properties of HPC electrode with mass loading of 21.2 mg·cm^-2^: (a) CV curves, (b) GCD curves, (c) Rate capability, (d,e) Nyquist plots, (f) cycling stability.


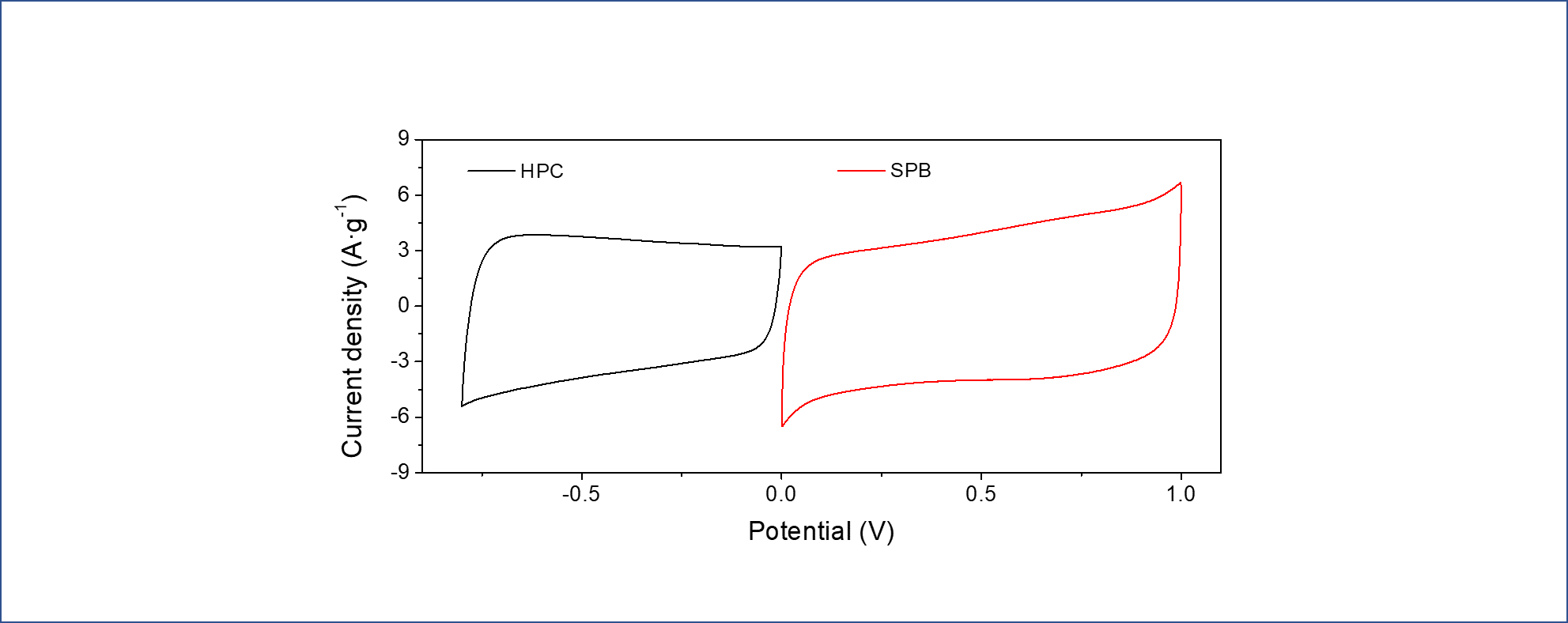


**Figure S22** CV curves of SPB electrode and HPC electrode at the scan rate of 20 mV·s^-1^.


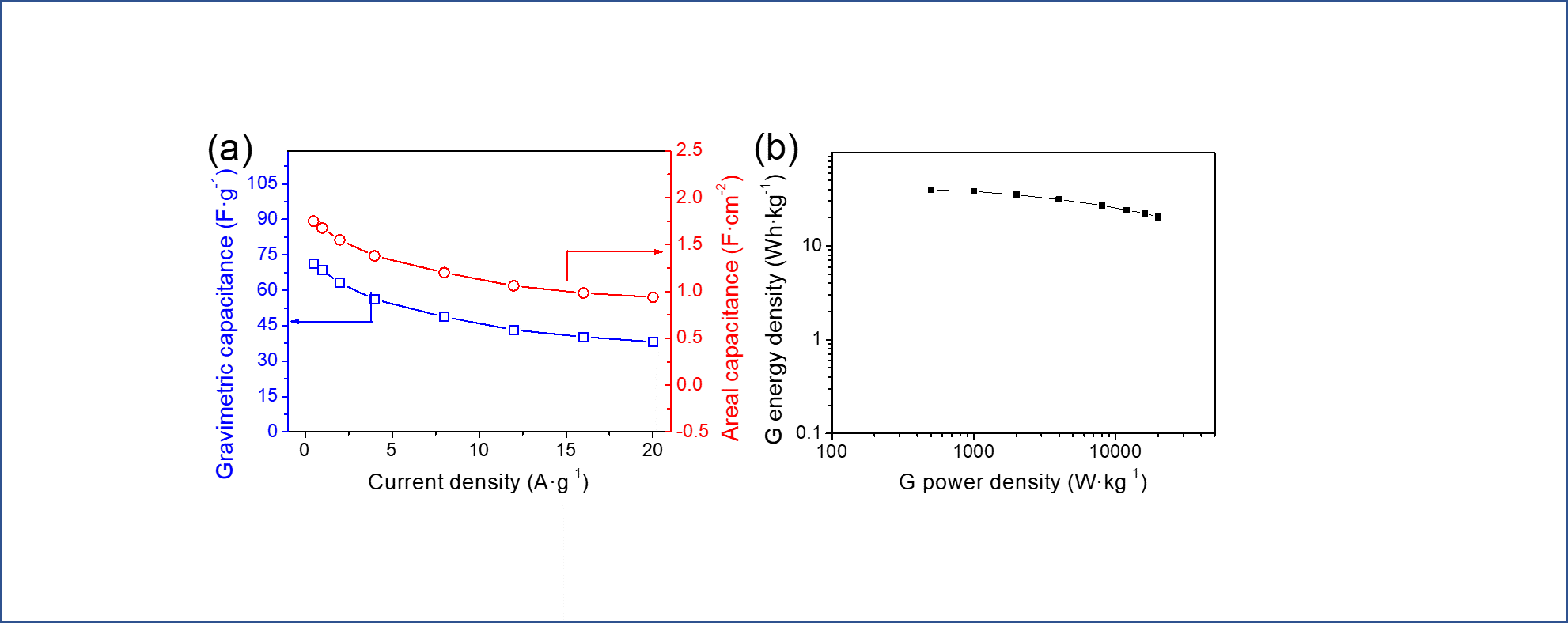


**Figure S23** Electrochemical properties of SPB/HPC device: (a) rate capability, (b) Ragone plots (G: gravimetric).

**Table S3** Electrochemical performance of MnO_2_-based high-mass-loading supercapacitors.

| **Supercapacitors**  **(Full cell)** | **Total mass**  **(mg·cm^-2^)** | **Voltage window** | **Specific capacitance**  **(F·g^-1^)** | **Areal energy density**  **(mWh·cm^-2^)** | **Electrolyte** | **Scan rate/**  **current density** | **Cycling**  **stability** | **Ref.** |
| --- | --- | --- | --- | --- | --- | --- | --- | --- |
| CDC@MnO_2_//CDC@Fe_2_O_3_ | 11.6 | 2.1V | unknown | 0.13 | PVA/LiClO_4_ | 1.0 mA·cm^-2^ | unknown | [9] |
| cellulose/f-CNT/MnO_2_-120 | unknown | 1.0 V | unknown | 0.25 | 1.0 M Na_2_SO_4_ | 1.0 mA·cm^-2^ | 79% (10000 cycles) | [22] |
| MnO_2_/CC//ACC | 16 | 2.0 V | unknown | 0.36 | 1.0 M Zn(NO_3_)_2_ | 1.0 mA·cm^-2^ | unknown | [31] |
| ACCC//MnO_2_ | unknown | 2.3 V | 55.2 | 0.36 | 1.0 M Na_2_SO_4_ | 0.5 mA·cm^-2^ | 91% (10000 cycles) | [32] |
| 3DG/MnO_2_ | unknown | 0.8 V | 66.4 | 0.39 | 3.0 M LiCl | 0.5 mA·cm^-2^ | 92.9% (20000 cycles) | [33] |
| MnO_2_/C | 17.3 | 2.0 V | unknown | 0.49 | 1.0 M Na_2_SO_4_ | 1.0 mA·cm^-2^ | 92.6% (10000 cycles) | [34] |
| GNF@NiCo_2_O_4_/MnO_2_//GNF@AC | unknown | 2.1 V | 61.2 | 0.5 | 1.0 M Na_2_SO_4_ | 2.0 mA·cm^-2^ | 82.4% (100 cycles) | [3] |
| WC@MnO_2_-20/WC@MnO_2_-20 | 20.8 | 1.0 V | 87 | 0.502 | 6.0 M KOH | 1.0 mA·cm^-2^ | 75.2% (10000 cycles) | [19] |
| N-MnO_2_//ACC | unknown | 2.0 V | 69 | 0.56 | 1.0 M Na_2_SO_4_ | 5.0 mA·cm^-2^ | 96% (8000 cycles) | [35] |
| PIC-CNTs-MnO_2_//PIC-CNTs | 13.9 | 2.0 V | 76.5 | 0.63 | 1.0 M Na_2_SO_4_ | 0.5 mA·cm^-2^ | 85% (5000 cycles) | [36] |
| [MnO_2_@CFCBSCA](mailto:MnO2@CFCBSCA) | unknown | 2.0 V | unknown | 0.7 | 1.0 M Na_2_SO_4_ | 2.3 mA·cm^-2^ | 80.8% (10000 cycles) | [37] |
| [N-PCH@WC-Mn](mailto:N-PCH@WC-Mn) | 35.6 | 1.0 V | 43.9 | 0.72 | 6.0 M KOH | 1.0 mA·cm^-2^ | 90.7% (10000 cycles) | [38] |
| MnOx/CCB/CNTs//APC APC/CCB/CNTs: | 35 | 2.0 V | 76.2 | 0.78 | 1.0 M Na_2_SO_4_ | 0.5 A·g^-1^ | 98.2% (8000 cycles) | [39] |
| MnO_2_-60//V_2_O_5_ NF | 21.6 | 2.0 V | 70.2 | 0.83 | 1.0 M Na_2_SO_4_ | 1.0 mA·cm^-2^ | >90% (8000 cycles) | [15] |
| Mo-MnO_2_@CC//C-Ti_3_C_2_T_x_@CC | 35.8 | 2.4 V | unknown | 0.89 | 1.0 M Na_2_SO_4_ | 2.0 mA·cm^-2^ | unknown | [40] |
| MnO_2_/HGF//PPy-NC/HGF | unknown | 2.0 V | unknown | 0.95 | PVA/LiCl | 2.0 mV·s^-1^ | 70.2% (5000 cycles) | [26] |
| **SPB/CCB/CNTs//HPC/CCB/CNTs** | **37** | **2.0 V** | **71.2** | **0.97** | **1.0 M Na_2_SO_4_** | **0.5 A·g^-1^** | **94.7% (10000 cycles)** | **This work** |


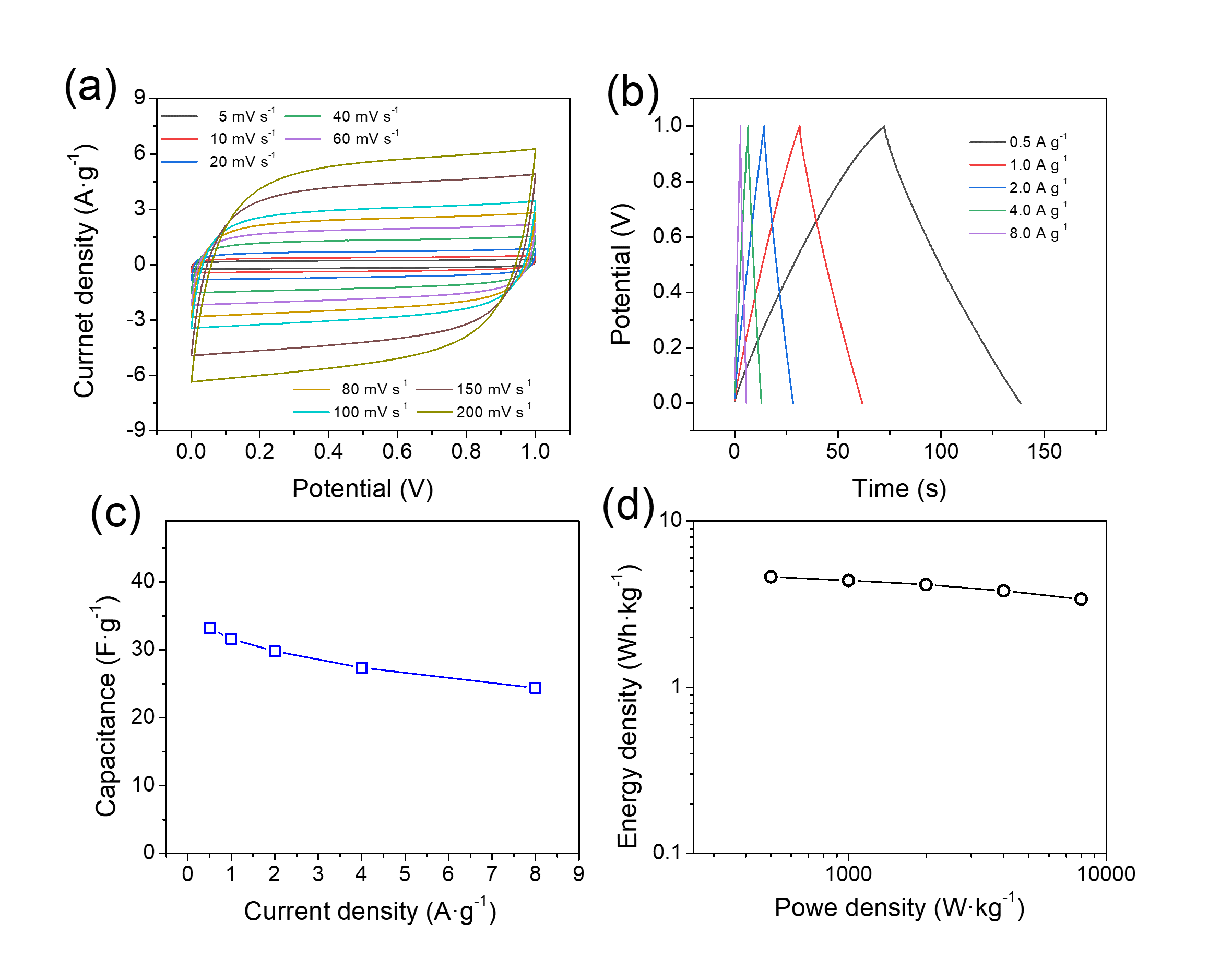


**Figure S24** Electrochemical properties of commercial porous carbon (BET SSA: 2000 m^2^·g^-1^) based supercapacitor: (a) CV curves, (b) GCD curves, (c) rate capability, (d) Ragone plots.

**Reference**

[1] K. Xiao, J. W. Li, G. F. Chen, Z. Q. Liu, N. Li, Y. Z. Su, *Electrochim. Acta* 149 (2014), 341-348.

[2] J. G. Wang, Y. Yang, Z. H. Huang, F. Kang, *J. Power Sources* 204 (2012), 236-243.

[3] J. Jin, J. Ding, X. Wang, C. Hong, H. Wu, M. Sun, X. Cao, C. Lu, A. Liu, *RSC Adv.* 11 (2021), 16161-16172.

[4] A. M. Abdelrahim, M. G. Abd El-Moghny, M. E. El-Shakre, M. S. El-Deab, *J. Energy Storage* 57 (2023), 106218.

[5] H. Wang, C. Xu, Y. Chen, Y. Wang, *Energy Storage Mater.* 8 (2017), 127-133.

[6] Y. Zhang, J. Fu, P. Cui, S. Cheng, X. Cui, T. Qin, J. Zhou, Z. Zhang, Q. Su, E. Xie, *Electrochim. Acta* 389 (2021), 138761.

[7] N. Wang, Y. Liu, B. Sun, Y. Wang, *J. Porous Materials* 29 (2022), 621-628.

[8] A. E. Fischer, K. A. Pettigrew, D. R. Rolison, R. M. Stroud, J. W. Long, *Nano Lett.* 7 (2007), 281-286.

[9] Y. Zhang, X. Yuan, W. Lu, Y. Yan, T.W. Chou, *Chem. Eng. J.* 368 (2019), 525-532

[10] N. R. Chodankar, S. J. Patil, G. S. Rama Raju, D. W. Lee, D. P. Dubal, Y. S. Huh, Y. K. Han, *ChemSusChem* 13 (2020), 1582-1592.

[11] Z. Yang, J. Ma, S. Araby, D. Shi, W. Dong, T. Tang, M. Chen, *J. Power Sources* 412 (2019), 655-663.

[12] L. Hu, W. Chen, X. Xie, N. Liu, Y. Yang, H. Wu, Y. Yao, M. Pasta, H. N. Alshareef, Y. Cui, *ACS nano* 5 (2011), 8904-8913.

[13] L. Lyu, K.D. Seong, J.M. Kim, W. Zhang, X. Jin, D.K. Kim, Y. Jeon, J. Kang, Y. Piao, *Nano-Micro Lett.* (2019), 12.

[14] Y. He, W. Chen, X. Li, Z. Zhang, J. Fu, *ACS nano* 7(2013), 174-182

[15] Huang, Zi-Hang, Song, Yu, Feng, Dong-Yang, Sun, Zhen, Xiaoqi, Liu, *ACS nano* 12(2018), 3557-3567.

[16] H. Huang, R. Chen, S. Yang, L. Li, Y. Liu, J. Huang, *High Perform. Poly.* 32(**2020)**, 286-295.

[17] W. Zeng, H. Quan, J. Meng, W. Wei, M. Liu, D. Chen, *Appl. Surf. Sci.* 572(2022), 151323

[18] B. J. Choudhury, V. S. Moholkar, *Ultrason. Sonochem.* 82 (2021), 105896.

[19] L. Chen, F. Wang, Z. Tian, H. Guo, C. Cai, Q. Wu, H. Du, K. Liu, Z. Hao, S. He, *Small* 18 (2022), 2201307.

[20] Y. Mao, J. Xie, C. Guo, H. Liu, H. Xiao, W. Hu, *Chem. Eng. J.* 426 (2021), 131188.

[21] Z. Pan, L. Jin, C. Yang, X. Ji, M. Liu, *Chem. Eng. J.* 470 (2023), 144084.

[22] J. P. Jyothibasu, R. H. Wang, K. Ong, J. H. L. Ong, R. H. Lee, *Cellulose* (2021), 1-19.

[23] Y. Wang, H. Quan, Q. Zhang, B. Tan, W. Chen, D. Chen, ACS Appl. Nano Mater. 24(2024), 27988–27997.

[24] S. Zhang, L. Li, Y. Liu, Q. Li, *Carbohyd. Polym.* 326 (2024), 121661.

[25] J. M. Jeong, S. H. Park, H.J . Park, S. B. Jin, S. G. Son, J. M. Moon, H. Suh, B. G. Choi, *Adv. Funct. Mater.* 31 (2021), 2009632.

[26] X. Xu, *Research* 2020 (2020), Article ID: 7304767

[27] C. Wallar, D. Luo, R. Poon, I. Zhitomirsky, *J. Materi. Sci.* 52 (2017), 3687–3696

[28] J. Wang, W. Guo, Z. Liu, Q. Zhang, *Adv. Energy Mater.* 13 (2023), 2300224.

[29] R. Chen, R. Poon, R.P. Sahu, I.K. Puri, I. Zhitomirsky, *J. Electrochem. Soc.* 164 (2017), A1673.

[30] Y. Zhou, X. Cheng, B. Tynan, Z. Sha, F. Huang, M.S. Islam, J. Zhang, A.N. Rider, L. Dai, D. Chu, *Carbon* 184 (2021), 504-513.

[31] Y. Wang, H. Quan, Q. Zhang, B. Tan, W. Chen, D. Chen, *ACS Appl. Nano Mater.* 7 (2024), 27988-27997.

[32] Y. Zhang, J. Fu, P. Cui, S. Cheng, E. Xie, *Electrochim. Acta* (2021), 138761.

[33] B. Yao, S. Chandrasekaran, J. Zhang, W. Xiao, F. Qian, C. Zhu, E.B. Duoss, C. M. Spadaccini, M.A. Worsley, Y. Li, *Joule* 3 (2019), 459-470

[34] Y. Guo, J. Chen, Z. Liang, X. Su, M. Sun, J. Tang, Z. Li, D. Dang, L. Yu, *J. Alloy. Compd.* 1010 (2025), 177125

[35] W. Zeng, H. Quan, J. Meng, W. Wei, M. Liu, D. Chen, *Applied Surface Science* 572 (2022), 151323.

[36] S. Zhu, T. Li, V.K. Bandari, O.G. Schmidt, M. Gruschwitz, C. Tegenkamp, M. Sommer, S. Choudhury, 13 (2021), 58486-58497

[37] C. Zhou, X. Wu, C. Luo, Z. Liu, L. Wang, C. Yang, H. Yu, *Susta. Mater. Techno.* 40 (2024), e00891

[38] F. Zhao, L. Lin, J. Zhang, J. Liu, *J. Energy Storage* 95 (2024), 112631

[39] S. Zhu, M. Sun, J. Jiang, X. Zhan, C. Du, C. Ding, D. Wei, X. Huang, *ACS Appl. Nano Mater.* 7 (2024), 28593-28601.

[40] Z. Pan, L. Jin, C. Yang, X. Ji, M. Liu, *Chem. Eng. J.* 470 (2023), 13.
